# Supplementary material for: Hippocampo-neocortical interaction as compressive retrieval-augmented generation
Source: Nat Commun. 2026 Jun 25;17:7971. doi: 10.1038/s41467-026-74357-6 (PMC13447824; doi:10.1038/s41467-026-74357-6)
Supplement: Supplementary file 1 [file 41467_2026_74357_MOESM1_ESM.pdf]

S1 Supplementary information

S1.1 Further model details

S1.1.1 Modelling sequence learning

Our simulations use autoregressive sequence models to represent the generative networks trained through hippocampal replay. Specifically transformer-based deep neural networks for text generation are used.

The primary goal during training is to adjust the model's parameters through maximum likelihood estimation, so that the probability it predicts for the true next item in each sequence, based on the items so far, is as high as possible. In other words, the network's weights are updated to predict the probability distribution of the next item as accurately as possible, a process known as causal language modelling. The training data for the original GPT-2 model is the WebText dataset of online content. Once the model is trained, it predicts the probability distribution across all items given the items so far. To generate a sequence one can sample from this distribution or simply take the most probable item at each step. The equation below gives the probability of a sequence x as a product of conditional probabilities of its items:

$$p(x) = \prod_{i=1}^{n} p(s_n | s_1, ..., s_{n-1})$$

We use the Transformers Python library (*1*) in all simulations. The first stage of using an LLM is to prepare the inputs with tokenisation. A tokeniser maps commonly occurring chunks of characters to IDs (in order to look up the right token embedding in a learned embedding matrix); in the case of language tokens are often words or parts of words. The concept of tokenisation is applicable to arbitrary sequences, but for simplicity and consistency across the simulations all stimuli are converted to strings of characters (if they are not already text-based). For Mistral 7B the same tokeniser is used as in the pre-trained model, but for the inference models a custom one is used, to ensure that relationships (e.g. EAST or CHILD_OF) and entities (e.g. 'xy') were each represented by a single token. (Without this, the default tokeniser does this inconsistently, e.g. with some pairs such as 'be' represented as one token because they commonly co-occur, and other such as 'bx' as two tokens because they do not.)

What exactly does causal language modelling with a custom dataset involve? First the training data is split into blocks, and then for every block the cross-entropy loss is aggregated across all the next token prediction tasks within the block. For example, for GPT-2 the block size (which is also the context size of the trained model) is 1024 tokens. This means that the model is trained to consider up to 1024 tokens of context when predicting the next token in a sequence. So for each block the model tries to predict the second token based on the first token, then the third token based on the first two, and so on, until it predicts the final token based on the preceding 1023.

The loss measures the difference between two probability distributions: the distribution predicted by the model and the actual distribution in the data. For each token prediction task, the actual distribution is a 'one-hot' vector with a one for the real next token and zeros elsewhere. Specifically, the cross-entropy loss for a single prediction task is calculated as the negative log probability assigned by the model to the actual next token. For a block of tokens, the total loss is the sum of the cross-entropy losses for each token prediction task within the block, and the weights of the model are updated based on this total loss. This procedure is the same whether the model is fine-tuned or trained from scratch.

The learning rate decreases over the course of training in all simulations, as per the default behaviour of the Trainer class in the Transformers Python library (*1*).

S1.1.2 Sampling options

There are many ways to generate sequences from a trained sequence model like Mistral 7B or GPT-2. The simplest is greedy decoding, where the model selects the token with the highest probability as the next token in the sequence. However this can lead to repetitive and predictable sequences. Sampling from the learned probability distribution with a given temperature introduces randomness into the selection of the next item, and provides a way to control the model's 'imaginativeness'. Specifically, the temperature parameter determines the 'sharpness' of the distribution from which output tokens are selected, so that sequences at a higher temperature are more 'imaginative', but more likely to be nonsensical.

The equation below describes the 'softmax with temperature' function that is applied to the vector of scores for each token to produce a vector of *probabilities*. A large temperature T flattens the distribution, whereas T close to zero approximates a 'one-hot' vector, with a probability of one for the most likely token:

$$\sigma(s_i) = \frac{e^{\frac{s_i}{T}}}{\sum_{j=1}^{n} e^{\frac{s_j}{T}}}$$

Unless stated otherwise, sequences are sampled from the probability distribution of tokens with a given temperature. Beam search is a search strategy used in some simulations, which considers multiple potential paths through the model's probability 'landscape'. At each step, it keeps a fixed number (the beam width) of the most probable sequences generated so far and extends them, eventually choosing the sequence with the highest overall probability.

S1.1.3 Further details on attention

In short, the attention mechanism works as follows (explained with reference to GPT-2): a query, a key, and a value vector are produced for each element in the sequence by learned weights. For a given element, the model computes a set of attention scores which determine how much attention should be paid to each element of the input sequence when representing this element. This is achieved by taking the dot products of the given element's query vector with the key vectors for each element. The final output is the sum of the value vectors for each element weighted by the corresponding attention scores (with some normalisation), allowing the model to aggregate information from the most relevant parts of the input. This describes a single 'attention head', but GPT-2's architecture features multiple attention blocks, each with multiple attention heads, which tend to develop different 'specialisms' through training. To be more precise the equation is given below:

$$\text{Attention}(Q, K, V) = \text{softmax}\left(\frac{QK^T}{\sqrt{d_k}}\right)V$$

In this equation, Q, K and V represent the query, key, and value matrices, respectively. These are derived from the input data. d_k represents the dimensionality of the keys (and queries), and is used to scale the dot products in a way that leads to more stable gradients. The softmax function is applied to ensure the weights sum up to 1. When the resulting term is matrix multiplied by V, the result is the relevance-weighted sum of the vector representations in V, for each token in V. In other words, the attention mechanism enriches a token's representations with a relevance-weighted sum of the other tokens' representations. See (*2*) for a detailed description of this process.

Self-attention is a special case of attention in which elements of a sequence 'attend to' the sequence they are a part of. Specifically, 'masked' self-attention is used, meaning that the representation of a token in an attention block only 'attends to' preceding tokens. Previous models like BERT (*3*) perform much less well at text generation partly because their attention is not masked in this way.

Transformer blocks come in different varieties. GPT-2 (*4*) is a decoder-only model consisting of a stack of transformer decoder blocks. Each such block consists of a masked self-attention layer followed by a

feedforward layer. In a nutshell, inference with GPT-2 works as follows: First an embedding for each token is obtained from a learned embedding matrix. A key point is that position embeddings representing the tokens' positions in the sequence are added to the token embeddings (these are required because self-attention is permutation-invariant, so otherwise information about the order of items in the sequence would be lost). Then the sequence of embeddings passes through a series of transformer decoder blocks, each further enriching the token representations so that they come to capture the meaning of the text. See (*5*) for helpful illustrations of the stages of processing in GPT-2. GPT-2 (*4*) comes in several sizes, with the number of transformer blocks depending on the size of the model. The small variant has 12 transformer blocks, the medium variant has 24 transformer blocks, and the large variant has 36 transformer blocks.

S1.1.4 Context compression comparison

Context compression has become an important problem in LLM research because performance often depends on access to relevant information, but context size is limited. Even when long-context models are available, this is computationally costly, and providing too much distracting information to the model can degrade performance.

Context compression techniques for LLMs can be divided into extractive and abstractive approaches, where the former selects a subset of the original context, and the latter summarises the context in some way. Extractive methods include LLMLingua (*6*), in which an auxiliary model predicts the importance of each token for a given LLM, and only the most important tokens are included in the context.

One intuitive form of abstractive context compression simply produces a textual summary of the input, e.g. see RECOMP (*7*). But abstractive methods can also map long contexts into compact latent representations. The in-context autoencoder approach (*8*) compresses arbitrarily long inputs into a fixed number of memory slots. The encoder's weights are trained in such a way that the LLM can reconstruct the original context conditioned only on the memory slots. Intriguingly, the authors find that the bigger and better the LLM, the more the context can be compressed at the same reconstruction accuracy.

The xRAG (*9*) method used here is similar to ICAE, but compresses the context to a single token. The primary reason for using xRAG rather than ICAE is because the stimuli in our simulations are short, and the pre-trained ICAE encoders are designed to compress contexts to 128 tokens, which can be longer than the stories themselves. However there is no strong theoretical reason to prefer xRAG to other candidate methods for abstractive context compression. One drawback of xRAG is that compressing texts to a single token then using many tokens to encode details is odd, as the core of the memory takes up a fraction of the capacity of the more extraneous details.

S1.1.5 Modelling sequence memory in the hippocampus

Here we provide further context to motivate the model of the hippocampus.

In the static model in (*10*), a modern continuous Hopfield network (MCHN; (*11*)) represents the hippocampus, interpreted such that the feature units activated by an event are bound together by a memory unit (*12*). In the sequential model, this could be adapted to store sequences as follows. Let us consider sequences represented as strings of characters or symbols, which can capture language, spatial trajectories, transitions in a graph, stimuli in sequential learning tasks, and more.

(*13*) give a unifying account of how neural network models of associative memory such as MCHNs operate, which helps to explain the extension to sequences. They develop the biologically plausible modern Hopfield network (*14*) into 'a general framework for understanding the operation of . . . memory networks as a sequence of three operations: similarity, separation, and projection', which they term 'universal Hopfield networks' ((*13*), Abstract). They observe that any of these models can be made asymmetric or heteroassociative rather than autoassociative, if the projections from feature units to memory units capture the current

state, but the projections from memory units back to feature units are different (for sequences, these projection weights would correspond to the 'next state'). Then one state retrieves the next, rather than each state retrieving a pattern-completed version of itself. That is, we want to store patterns X and 'next patterns' X_{next} instead of just patterns, and change the MCHN's update rule accordingly:

$$\xi_{next} = X_{next} softmax(\beta X^T \xi)$$

The modern continuous asymmetric Hopfield network (MCAHN) converts the MCHN to work for sequences, consistent with the framework above (*15*). However there is a problem when you try to apply this naively to certain sequences, as the MCAHN is by default a 'Markov chain' model of sequential memory. Whilst it works for sequences in which items are unique, it doesn't work for, e.g., sentences without modification, as it cannot accurately remember sequences with repeated items; see Figure 5a of (*16*). When an item is repeated in a sequence, (*16*) show that a MCAHN has trouble recalling the next item, instead producing a composite.

(*16*) introduce the temporal predictive coding network (tPC), a more complex network learnt by gradient descent, based on the intuition that higher layers minimize prediction error at lower layers (*17*). (A drawback of the tPC is that the model must be 'presented with the sequence for multiple epochs' until convergence, so it is no longer capable of one-shot learning, which arguably undermines its plausibility as a model of hippocampal encoding.) They show that the one-layer tPC also has the MCAHN's issue with repeated items, but suggest the two-layer tPC to resolve this. As (*16*) show in Figure 5d of their paper, the hidden layer represents an item's position in the sequence so the next item can be recalled correctly.

Alternatively, one could combine the concept of the modern asymmetric Hopfield network (with return projections from memory to feature units representing the next state) with a state representation that captures the history. This is based on previous work involving the role of temporal context in memory (*18, 19*). Consider the example of encoding sentences, i.e. sequences of characters. Suppose each state is a vector of length equal to the number of symbols, consisting of one at the index of the current symbol, plus the previous state multiplied by some decay factor. See Figure S1 for an example. This has the benefit of still being compatible with one-shot learning, although the memory capacity may not scale as well as the more complex predictive coding model approach. Initial testing suggests that this model can encode and retrieve sentences (although retrieval performance goes down with sentence length).

To show how the preceding 'context' is captured, consider the example of encoding sentences, i.e. sequences of characters. Each state can be represented by a vector of length equal to the number of symbols, with one at the index of the current symbol, plus the previous state multiplied by some decay factor. In other words, we want a vector representation $\mathbf{x_i}$ of the ith state which is a sum of the current symbol's vector $\mathbf{v}$ and activity from the previous state $\mathbf{x}_{i-1}$ multiplied by a decay rate λ (noting that the states are then normalised to unit length):

$$\mathbf{x}_i = \frac{\mathbf{v}_i + \lambda \cdot \mathbf{x}_{i-1}}{||\mathbf{x}_i||}$$

As with all modern Hopfield network (*11*) variants, the inverse temperature, β, is a key parameter determining the network's behaviour. In this case a high value of β would be desirable to avoid composite states. The softmax function is given below; in the limit of high β, the output of this function becomes a vector with one at the index of the maximum and value and zero elsewhere:

$$softmax(z_i) = \frac{e^{\beta z_i}}{\sum_{j=1}^{K} e^{\beta z_j}}$$

Episodic memories consist of complex representations at each time step, with autoassociative connections thought to reconstruct the current time step, and heteroassociative connections to activate the next (see (*20*)).

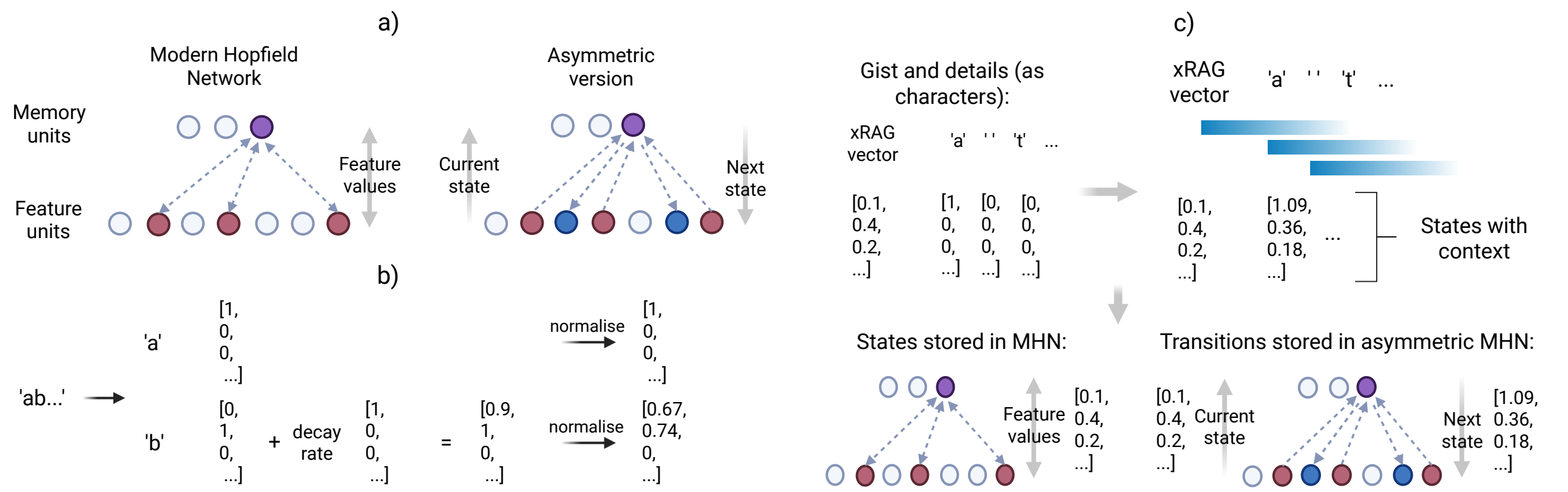

Figure S1: Using modern Hopfield networks (MHNs) to model the hippocampus. a) In asymmetric versions of MHNs, the weights between feature and memory units differ, as visualised from the perspective of (*12*). b) A sequence of arbitrary symbols, e.g. 'ab...', is represented as a sequence of vectors over the feature units, where each vector is the sum of the current state plus the decay rate (here set to 0.9) times the previous state. c) To store a narrative in compressed form, the xRAG vector and details are represented as a series of states, with a decaying trace of previous states. The states are encoded in an MHN, and the state-next state transitions in an asymmetric MHN. In the latter, for each state the return connections from memory units to feature units encode the next state.

As described in 'Modelling the hippocampus', the asymmetric MHN described above is combined with a 'standard' autoassociative MHN for retrieving an item from a noisy query.

Whilst this simple model helps justify the assumption that the hippocampus can store sequences associatively, it clearly has some limitations. For example, it suggests that when a state from a certain point in an encoded sequence triggers recall, states preceding that point cannot be recalled (only the subsequent states). This could be addressed by adding heteroassociative connectivity backwards in time. A more fundamental problem is that the cue for retrieval can be a noisy version of the whole sequence, but in this simple model retrieval begins by denoising a single item in isolation. This means that retrieval may perform poorly when all items are so noisy that retrieval of any item individually is poor, but the sequence as a whole is sufficient to identify the right memory. Future work could investigate biologically plausible models of hippocampal memory that address this.

S1.2 Supplementary results

S1.2.1 Supplementary figures

Figure S1 illustrates the model hippocampus. Figure S2 shows some additional results relating to the model hippocampus.

Figure S3 shows the results of performing principal component analysis on token representations in the spatial inference model, across all layers of the model.

S1.2.2 Supplementary tables

Table S1 shows examples of encoded and recalled stories from 'Modelling hippocampal-neocortical interaction as retrieval-augmented generation'.

Tables S2, S3, and S4 show that the patterns in concreteness, specificity, and richness in detail hold even when controlling for length. This is important because for both the HIPPOCORPUS and simulated data, there

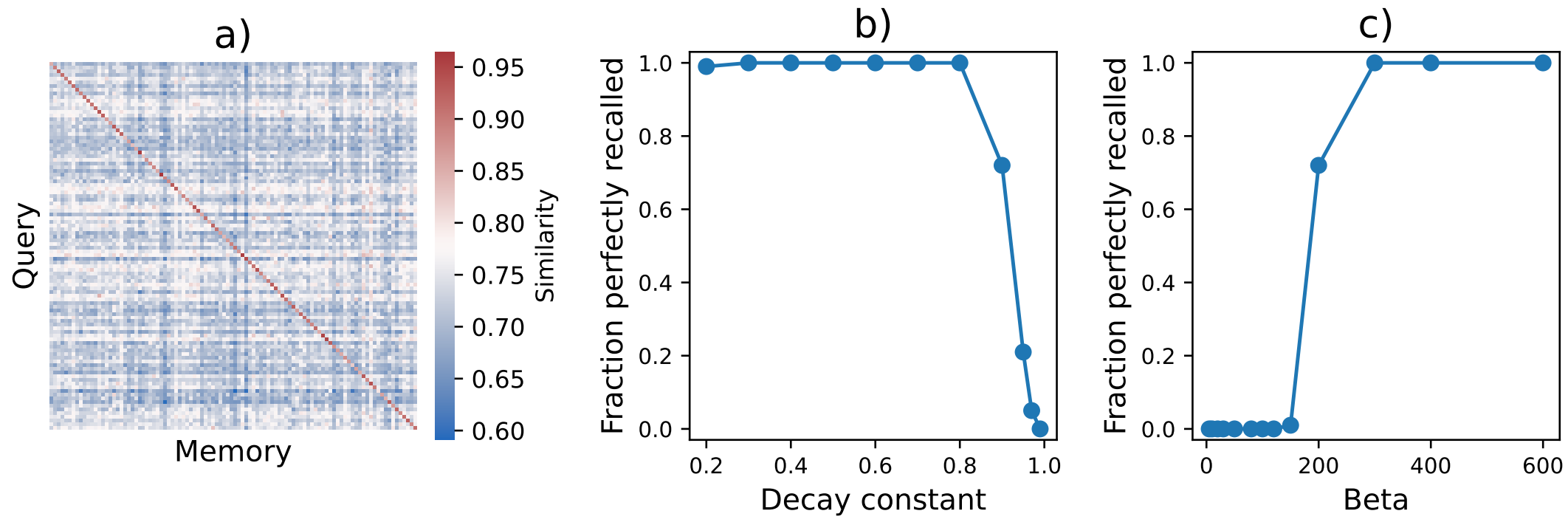

Figure S2: Modelling the hippocampus with a dual-MHN model for 100 stories from (*21*). a) Similarity (dot product) between each xRAG query embedding (constructed from an 80-character excerpt) and the stored xRAG story embedding (whole story), showing a strong diagonal indicating correct memory selection. b) Fraction of episodes perfectly recalled as a function of the decay constant. c) Fraction of episodes perfectly recalled as a function of inverse temperature β (applied identically to both MHNs), showing improved recall with sharper retrieval. Source data are provided as a Source Data file.

are significant Pearson correlations between length and each attribute (Table S2). For the HIPPOCORPUS data, regressing out the length, and then comparing the residual score for each attribute (i.e. the component which cannot be explained by the length) using a t-test, gives the results in Table S3. Doing the same for the simulated data gives the results in Table S4.

Table S5 shows the Bartlett story recalled at different temperatures, illustrating the approximate memorisation of the narrative in 'Gist-based distortions and prior experience'. Tables S6 and S7 give the inference accuracy for each template tested in 'Neocortical learning of sequential structure for problem solving'.

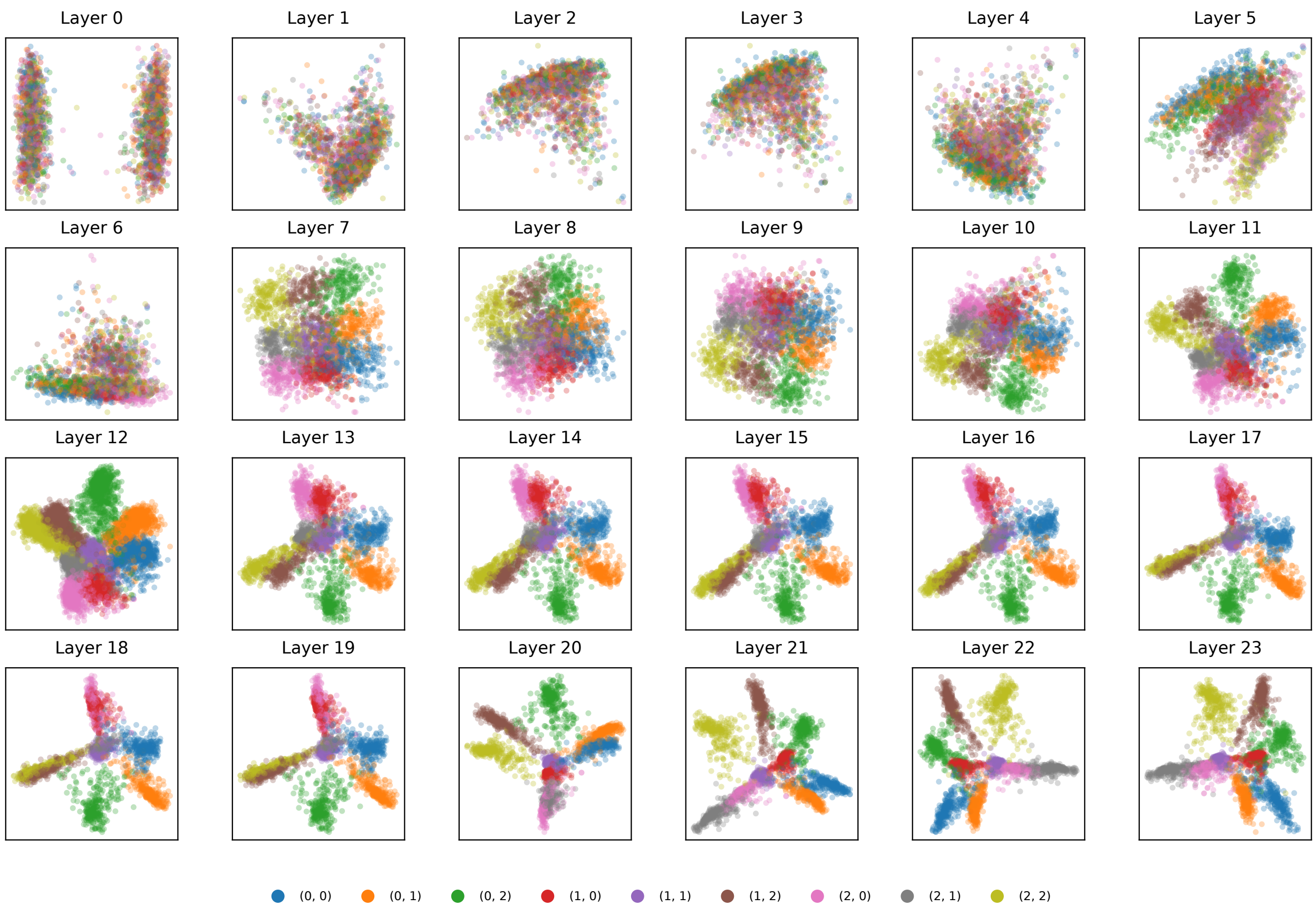

Figure S3: Principal component analysis of token representations in *all* layers of the spatial inference model in 'Neocortical learning of sequential structure for problem solving' (see Figure 7). Activations for each token of random walks in many different environments were extracted from a given layer of the model, to obtain an average vector for each location. Each subplot shows these representations projected into 2D with principal component analysis, colouring each location by its co-ordinates in the environment so that points with the same colour are different nodes which occupy the same position in the 3x3 grid. Source data are provided as a Source Data file.

Original	Gist only	Stored detail	Gist + detail
Carlton is playing soccer. His team is down by 2 goals. He goes in the zone. He scores 3 goals to win the game. Carlton is carried off the field as a champion.	Carlton scored a goal, but the other team scored a goal right after. Carlton was feeling down, but his teammates cheered him up and encouraged him to keep trying. Carlton scored another goal, and his team won the game.	He scores 3 goals to win the game	Carlton is playing soccer in the final game of the season. He is down 2-0, but he doesn't give up. He scores a goal to make it 2-1. Then, he scores another goal to make it 2-2. Finally, he scores the winning goal in overtime to win the game for his team. The crowd goes wild and Carlton is a hero.
Monica woke up with pain in her right ear. She went to the doctor. The doctor looked in her ears with an otoscope. The doctor informed Monica she had an ear infection. The doctor wrote Monica a prescription for antibiotics.	Monica woke up with pain in her right ear. She went to the doctor and described her symptoms. The doctor examined her ear and diagnosed her with an ear infection. The doctor prescribed antibiotics and advised Monica to rest and avoid loud noises. Monica followed the doctor's instructions and felt better after a few days.	The doctor looked in her ears with an otoscope	Monica woke up with pain in her right ear. She went to the doctor and the doctor looked in her ears with an otoscope. The doctor diagnosed Monica with an ear infection and prescribed her antibiotics. Monica took the antibiotics for a week and her ear pain went away.
Charles had always wanted to have a college degree. He worked hard for many years to complete his courses. Finally he turned in his last college essay. All of his family attended his college graduation. Charles was proud that he had a college degree.	Charles worked hard and went to college. He studied very hard and worked multiple jobs to pay for his education. He graduated with a degree in English. He was very proud of his accomplishment and felt that it was a great feeling to finally have a college degree.	Finally he turned in his last college essay	Charles had always wanted to have a college degree. He worked hard and studied every day. He took classes, did his homework, and participated in class. He wrote many college essays and finally turned in his last one. He was proud of his accomplishments and was excited to graduate. He received his degree and was happy that he had achieved his goal.

Table S1: Narrative examples of retrieval-augmented generation with compression as a model of hippocampal-neocortical interaction, using Mistral-7B-Instruct-v0.2 plus xRAG weights to simulate neocortex. The 'Original' column gives the story 'experienced' by the system. The 'Gist only' column gives the reconstruction from just the xRAG vector, i.e. when the xRAG vector is retrieved from the hippocampus and used to condition the generative network. The 'Stored detail' column lists the most surprising detail given the xRAG vector. Specifically, all phrases are extracted from the text, then the perplexity of each phrase given the xRAG vector is calculated. In other words, for each phrase, the model is prompted with the xRAG vector and the phrase in question. Then the phrases are ranked in descending order of perplexity, with the top phrase used as the 'Stored detail'. The text embedding (from which the xRAG vector is derived) and details for each memory are encoded together in the model hippocampus. The 'Gist + detail' column gives the reconstruction of the memory from this representation.

Dataset	Concreteness	Richness in detail	Specificity
HIPPOCORPUS	$r = 0.328$, $p = 1.95 \times 10^{-171}$	$r = 0.525$, $p = 4.51 \times 10^{-482}$	$r = 0.410$, $p = 3.07 \times 10^{-276}$
Simulated	$r = 0.261$, $p = 1.68 \times 10^{-32}$	$r = 0.344$, $p = 1.12 \times 10^{-56}$	$r = 0.211$, $p = 1.17 \times 10^{-21}$

Table S2: Pearson correlations between story length and each LLM-derived attribute.

Attribute	Recalled vs. Retold	Recalled vs. Imagined	Retold vs. Imagined
Concreteness	$t = 3.854$, $p = 1.19 \times 10^{-4}$	$t = 9.260$, $p = 2.86 \times 10^{-20}$	$t = 3.884$, $p = 1.05 \times 10^{-4}$
Richness in detail	$t = 3.345$, $p = 8.33 \times 10^{-4}$	$t = 8.780$, $p = 2.15 \times 10^{-18}$	$t = 3.764$, $p = 1.71 \times 10^{-4}$
Specificity	$t = 4.690$, $p = 2.86 \times 10^{-6}$	$t = 8.471$, $p = 3.09 \times 10^{-17}$	$t = 2.281$, $p = 0.0226$

Table S3: The results of Welch's t-tests (two sided) on length-residualised attribute scores for HIPPOCORPUS stories.

Attribute	Original vs. Encoded	Original vs. Consolidated	Encoded vs. Consolidated
Concreteness	$t = 2.328$, $p = 0.0203$	$t = 6.526$, $p = 1.66 \times 10^{-10}$	$t = 4.630$, $p = 4.68 \times 10^{-6}$
Richness in detail	$t = 2.818$, $p = 0.00503$	$t = 6.897$, $p = 1.61 \times 10^{-11}$	$t = 4.762$, $p = 2.52 \times 10^{-6}$
Specificity	$t = 4.173$, $p = 3.55 \times 10^{-5}$	$t = 7.764$, $p = 4.69 \times 10^{-14}$	$t = 4.054$, $p = 5.83 \times 10^{-5}$

Table S4: The results of paired t-tests (two-sided) on length-residualised attribute scores for simulated stories.

Type	Recalled story example
Original	One night two young men from Egulac went down to the river to hunt seals and while they were there it became foggy and calm. Then they heard war-cries, and they thought: "Maybe this is a war-party". They escaped to the shore, and hid behind a log. Now canoes came up, and they heard the noise of paddles, and saw one canoe coming up to them. There were five men in the canoe, and they said: . . .
Temperature of 0	One night two young men from Egulac went down to the river to hunt seals and while they were there it became foggy and calm. Then they heard war-cries, and they thought: "Maybe this is a war-party". They escaped to the shore, and hid behind a log. Now the war-party started, and they heard the noise of paddles, and saw one canoe coming up to them. There were five men in the canoe, and they said: ...
Temperature of 0.5	One night two young men from Egulac went out to the river to hunt seals and while they were there they were robbed by the men from Egulac. The men ran back to Egulac and hid behind a log. When they came out they discovered a canoe coming up to them and they jumped up and began to fight. The canoe came down to the ground and they began to wrestle. The crowd cheered for the young men of Egulac. . . .
Temperature of 1	One night two young men from Egulac went up to the river and they saw a canoe coming up to them. Then they heard war-cries, and they heard the sound of paddles, aand they heard war-cries, and they saw one canoe coming up to them. It was full speed, and when it arrived the men said: "This is a war-party. We have come to conquer you in a fight". When they reached the shore, they saw one canoe . . .

Table S5: Recalled stories for different temperatures, for a model trained for five epochs with the 'Sports' category of Wikipedia data (*22*) as the background data distribution, and prompted with 'One night two young men from Egulac'.

**Inference template**	**Mean accuracy**
{} EAST {} WEST {}	1.0
{} WEST {} EAST {}	1.0
{} NORTH {} SOUTH {}	1.0
{} SOUTH {} NORTH {}	1.0
{} EAST {} SOUTH {} WEST {} NORTH {}	1.0
{} SOUTH {} WEST {} NORTH {} EAST {}	1.0
{} WEST {} NORTH {} EAST {} SOUTH {}	1.0
{} NORTH {} EAST {} SOUTH {} WEST {}	1.0
{} EAST {} EAST {} NORTH {} WEST {} WEST {} SOUTH {}	1.0
{} NORTH {} NORTH {} WEST {} SOUTH {} SOUTH {} EAST {}	1.0
{} EAST {} EAST {} SOUTH {} SOUTH {} WEST {} WEST {} NORTH {} NORTH {}	1.0
{} SOUTH {} SOUTH {} WEST {} WEST {} NORTH {} NORTH {} EAST {} EAST {}	1.0

Table S6: Spatial task inference templates and their average accuracies.

**Inference template**	**Mean accuracy**
{} CHILD_OF {} PARENT_OF {}	1.0
{} PARENT_OF {} CHILD_OF {}	1.0
{} GRANDCHILD_OF {} GRANDPARENT_OF {}	1.0
{} GRANDPARENT_OF {} GRANDCHILD_OF {}	1.0
{} CHILD_OF {} CHILD_OF {} PARENT_OF {} PARENT_OF {}	1.0
{} PARENT_OF {} PARENT_OF {} CHILD_OF {} CHILD_OF {}	1.0
{} CHILD_OF {} SPOUSE_OF {} SPOUSE_OF {} PARENT_OF {}	1.0
{} PARENT_OF {} SPOUSE_OF {} SPOUSE_OF {} CHILD_OF {}	1.0
{} CHILD_OF {} SPOUSE_OF {} CHILD_OF {} SPOUSE_OF {} GRANDPARENT_OF {} SIBLING_OF {}	0.99
{} GRANDPARENT_OF {} SIBLING_OF {} CHILD_OF {} SPOUSE_OF {} CHILD_OF {} SPOUSE_OF {}	1.0

Table S7: Family tree task inference templates and their average accuracies.

References

1. T. Wolf, *et al.*, HuggingFace's transformers: State-of-the-art natural language processing. *arXiv preprint arXiv:1910.03771* (2019).

2. J. Alammar, The Illustrated Transformer (2018), `https://jalammar.github.io/illustrated-transformer/`, accessed on March 19, 2024.

3. J. Devlin, M.-W. Chang, K. Lee, K. Toutanova, Bert: Pre-training of deep bidirectional transformers for language understanding. *arXiv preprint arXiv:1810.04805* (2018).

4. A. Radford, *et al.*, Language models are unsupervised multitask learners. *OpenAI blog* **1** (8), 9 (2019).

5. J. Alammar, The illustrated GPT-2 (2019), `https://jalammar.github.io/illustrated-gpt2/`, accessed on March 19, 2024.

6. H. Jiang, Q. Wu, C.-Y. Lin, Y. Yang, L. Qiu, LLMLingua: Compressing prompts for accelerated inference of large language models. *arXiv preprint arXiv:2310.05736* (2023).

7. F. Xu, W. Shi, E. Choi, Recomp: Improving retrieval-augmented lms with compression and selective augmentation. *arXiv preprint arXiv:2310.04408* (2023).

8. T. Ge, *et al.*, In-context autoencoder for context compression in a large language model. *arXiv preprint arXiv:2307.06945* (2023).

9. X. Cheng, *et al.*, xrag: Extreme context compression for retrieval-augmented generation with one token. *arXiv preprint arXiv:2405.13792* (2024).

10. E. Spens, N. Burgess, A generative model of memory construction and consolidation. *Nature Human Behaviour* pp. 1–18 (2024).

11. H. Ramsauer, *et al.*, Hopfield networks is all you need. *arXiv preprint arXiv:2008.02217* (2020).

12. D. Krotov, J. Hopfield, Large associative memory problem in neurobiology and machine learning. *arXiv preprint arXiv:2008.06996* (2020).

13. B. Millidge, T. Salvatori, Y. Song, T. Lukasiewicz, R. Bogacz, Universal Hopfield networks: A general framework for single-shot associative memory models, in *International Conference on Machine Learning* (PMLR) (2022), pp. 15561–15583.

14. D. Krotov, J. J. Hopfield, Dense associative memory for pattern recognition. *Advances in neural information processing systems* **29** (2016).

15. H. T. Chaudhry, J. A. Zavatone-Veth, D. Krotov, C. Pehlevan, Long sequence Hopfield memory. *arXiv preprint arXiv:2306.04532* (2023).

16. M. Tang, H. Barron, R. Bogacz, Sequential Memory with Temporal Predictive Coding. *arXiv preprint arXiv:2305.11982* (2023).

17. T. Salvatori, *et al.*, Associative memories via predictive coding. *Advances in Neural Information Processing Systems* **34**, 3874–3886 (2021).

18. N. Burgess, G. J. Hitch, Memory for serial order: A network model of the phonological loop and its timing. *Psychological review* **106** (3), 551 (1999).

19. M. W. Howard, M. J. Kahana, A distributed representation of temporal context. *Journal of mathematical psychology* **46** (3), 269–299 (2002).

20. H. Sompolinsky, I. Kanter, Temporal association in asymmetric neural networks. *Physical review letters* **57** (22), 2861 (1986).

21. N. Mostafazadeh, *et al.*, A corpus and cloze evaluation for deeper understanding of commonsense stories, in *Proceedings of the 2016 Conference of the North American Chapter of the Association for Computational Linguistics: Human Language Technologies* (2016), pp. 839–849.

22. T. Ziadé, Wikipedia Topics, `https://huggingface.co/datasets/tarekziade/wikipedia-topics` (2024), accessed: 2024-05-31.
