# Supplementary material for: Hippocampo-neocortical interaction as compressive retrieval-augmented generation
Source: Nat Commun. 2026 Jun 25;17:7971. doi: 10.1038/s41467-026-74357-6 (PMC13447824; doi:10.1038/s41467-026-74357-6)
Supplement: Supplementary file 3 [file 41467_2026_74357_MOESM3_ESM.pdf]

nature portfolio

Peer Review File

Hippocampo-neocortical interaction as compressive retrieval-augmented generation

Corresponding Author: Dr Eleanor Spens

This file contains all reviewer reports in order by version, followed by all author rebuttals in order by version.

Version 0:

Reviewer comments:

Reviewer #1

(Remarks to the Author)
This paper uses GPTs as models of hippocampal-cortical interactions during consolidation and retrieval. The simulations demonstrate how prior knowledge can bias memory recall, how information can be generalized over the course of consolidation, and how hippocampal memory retrieval can provide useful context for cortical prediction. This is, to my knowledge, the first systematic attempt to map components / functions of GPTs to memory systems and their interactions. There are several interesting insights, and I think the paper will make a highly valuable contribution to the literature. I expect many others will be interested in adapting and building on this framework.

I reviewed a version of this paper at a prior journal and was already very positive about it then. This version has focused on a subset of the simulations and added some depth to these. My original suggestions have already been incorporated and this version does not raise any new concerns for me.

(Remarks on code availability)

Reviewer #2

(Remarks to the Author)
This paper proposes a computational model explaining how the hippocampus (which stores episodic memories) and the neocortex (which stores general knowledge or semantic memory) work together. They model this interaction using ideas borrowed from machine learning, specifically retrieval-augmented generation (RAG) — a technique used in large language models (LLMs) like Chat GPT. More precisely, the model hypothesizes: 1) the neocortex acts as a generative model trained to predict experiences like an LLM for text processing; 2. the hippocampus stores episodic memories in a compressed form consisting of gist sequence plus surprising details; 3. the neocortex learns from hippocampal replays, extracting general patterns (semantic memory); 4. the hippocampus retrieves relevant memories, which in turn guide the neocortical model to generate coherent responses, like retrieval-augmented generation (RAG) in AI systems. The authors compared the model's performance with that of simpler models in AI as well as humans in spatial and narrative memory tasks. Based on these results, the authors suggest that the hippocampal-neocortical network functions similarly to AI foundation models that learn through prediction error minimization.

I reviewed the manuscript, assisted by a postdoc in my lab. We found that some results are interesting but also have several concerns about the manuscript. In short, we feel the manuscript is not clear enough and confusing, although neither of us is an expert in AI research, and we might have missed some essential points about the results. Below, we list our comments.

Major comments:

1. We were first excited to read the manuscript, but our enthusiasm dropped rapidly as we proceeded. The model uses two successful models in modern machine learning, Chat-GPT2 or Mistral 7B for the neocortex, which was trained on sequences representing hippocampal replay activities. The trained model exhibited impressive performance in various memory tasks. However, in a sense, the model's high performance is guaranteed from the beginning, as we already know the component models perform their own businesses excellently. However, the combined model will not be the best, as the

component models are not the state-of-the-art models. Therefore, from machine learning point of view, the manuscript does not seem to show sufficiently novel results. (However, if this study does show novel results in ML, the results should be explained.) On the other hand, the model is not strongly grounded in biological neural networks of the hippocampus and prefrontal cortex, although it explains some psychological phenomena observed in episodic memory. The authors are aware of the lack of neural-level support for the model (as they stated in the discussion), but we are still left with a doubt. After all, what novel points have we learned from this model about the hippocampal-neocortical mechanism of memory processing?

2. As many readers in neuroscience will only poorly know the machine learning models, especially Mistral 7B, the authors should explain the structure and function of these models in somewhat detail. In addition, we wonder whether the authors actually modeled the hippocampus or merely used gist sequences and surprising details (generated by the neocortex?) as its replay activity.

3. If we understand correctly, the most essential hypothesis of the model is that the hippocampal-neocortical neural system works as retrieval-augmented-generation (RAG) proposed in ref. 31. Is the present model just a biological implementation of the original model? Alternatively, does it offer a novel function that was missed in the original model? The author should argue for experimental evidence supporting this assumption, if any.

4. The model is meant to give insight into hippocampal-neocortical interactions, however, it is hard to understand the actual role of the hippocampus in the model. Figure 1a) shows State n and State n+1 with some illustration, which is not explained in the caption. Similarly, what the States here represent, and why only n and n+1 is included.

5. Figure 1, which should provide a clear overview of the model, looks very dense and becomes difficult to understand. Some things remain unexplained. If these things are not necessary, we would recommend leaving them out of the Figure to avoid confusion. Alternatively, they should be explained in the caption. For example, explanation for 1c) only states "The neocortical and hippocampal networks can work together to recall memories and solve problems via RAG.", but the meaning "New task" is unclear. The meaning of the color scheme is also not clear.

6. According to Fig. 1b, the hippocampus stores sequences in a compressed form in gist sequences and surprise details of specific episodes. Table 1 shows examples of the stories retrieved from the gist and detail of the hippocampal representations. However, the gist-only stories are more complex than the original ones in these examples, and they do not clarify how stories are decomposed into gist and detail by the hippocampus. Some examples of explicit hippocampal representations will be useful for readers unfamiliar with LLMs, although I am unsure whether showing such representations is possible.

7. In Fig. 3a, the authors showed a trade-off between efficiency and accuracy of memory recall. How do the hippocampus and neocortex differently contribute to efficiency and accuracy? Which component is more critical to the trade-off? What does "simply imagining the event" mean?

8. In Fig. 3b and c, the meaning of Original and Encoded is not explained. Is similarity the cosine similarity in the embedding space? This should be stated somewhere. Similarity of both "Encoded" and "Original" is increasing. Which one is the neocortex's reconstruction?

9. In Fig. 4g and 4h, the authors compared narrative distortion in memory recall between humans and the model. For short delays, both agents exhibited similar tendencies in making omission and extension errors. In contrast, for long delays, the two error types display largely different proportions in humans but similar proportions in the model. Please explain why so, if possible.

10. Spatial navigation is the most extensively studied subject in the research of the rodent hippocampus. Figure 6d presents the results of PCA of the representations extracted from different environments, and the authors demonstrated that the model outperforms pre-trained GPT-2 without a neocortex circuit. However, since the spatial environments studied here are simple, it remains unclear to what extent the results confirm the model's excellent performance in spatial navigation. For instance, can the model learn a shortcut from sample trajectories that do not involve it? What are gist sequences and surprise details in spatial navigation?

11. Related to comment 10, the baseline model (pretrained GPT-2) does not produce a topographic map of the environment, while "our model" does (Fig. 6a-d). Please explain the reason for this difference. Intuitively, Chat-GPT or Transformer-based LLM is thought to learn relationships between inputs based on their similarities. Therefore, I do not understand why GPT-2 alone cannot learn similarity between spatial locations.

12. Indeed, section 2.3 and Figure 6 are quite confusing. Please explain what "our model" refers to in Fig. 6.

Minor comments:

13. Some panels, for instance, those in Figs. 3 and 4 are referenced in a random order in the main text. Although I do not think this is unacceptable, it decreases the clarity of the manuscript.

14. It would be helpful to use colors consistently. For example in Figure 3, orange represents Memory size (a), "Encoded" (b and c), "Consolidated" (d), "Richness in detail" (e), "retold" (vs recalled) (g), Pre-consolidation word distribution (i). This makes it more difficult to quickly orient in the figures.

(Remarks on code availability)

Reviewer #3

(Remarks to the Author)
This manuscript starts off from a very reasonable and widely shared perspective on the involvement of the hippocampus and neocortex in memory formation, retrieval/regeneration and consolidation, and develops it using machine learning and in particular large learning models. Importantly, in such a perspective episodic memories are not retrieved in the same exact form they were originally stored but are, instead, the result of a generative process which reflects schemas previously consolidated in the neocortex. This "semanticization" accrues progressively, leading to increasing deviations, also in the concrete example offered in the manuscript of short 3-4 sentence "stories", from the original.

Specifically, the authors consider that memory consolidation can be modeled as a generative network with Retrieval-Augmented Generation (RAG). In this framework, the neocortex is represented as a pretrained large language model (Mistral 7B at first, later GPT-2 Medium), while the hippocampus functions both as a prompter and as a retrieval database that encodes context, the gist and surprising detail of an episode. The interaction between the two brain regions is thus assimilated to a Machine Learning method (RAG) that augments any given prompt to the LLM with relevant context from the prompter/database.

Applying RAG iteratively is taken to model memory consolidation: the neocortical "generator" is gradually trained on context-augmented prompts from the hippocampal "retriever".

Prompts take the form of:
[INST] Refer to the background document and answer the question.
Background: {XRAG TOKEN}
Question: {FIRST LINE} What happened (in detail)?
Other details to include: {SURPRISING DETAILS}. [/INST] The answer is:

This is all fine and good. Except that the development of the general perspective which is offered in the manuscript is not science, in our view, but rather an engineering demonstration. Assertions and hypotheses – which by and large we would happily subscribe to – are not mathematically proven or empirically tested and validated but rather exemplified with a reduced scale toy model and put forward as cartoon figures. It is in fact not entirely clear what is reduced in scale, here, since no actual neural circuit is simulated, although probably the 500 episodic strings can be regarded as a reduced scale of the experiences concurrently held in memory by a human being (we write "human" because of the focus in the manuscript on language and linguistic episodes).

A major concern for us is also that the authors could have more clearly articulated the conceptual logic of the RAG-inspired consolidation model, independent of the machine learning implementation. This would separate neuroscience talk from machine learning talk and could help highlight what is theoretically novel and what is not. It could perhaps be summarized as follows:

1. A sequential input from working memory (a story) is compressed as gist plus detail, in the hippocampus.

2. The first few sequences from working memory are presented to the neocortex which predicts the following sequences.

3. The neocortex then minimizes the prediction error between the compressed sequential input and its prediction and updates its weights.

4. Now the prediction is compressed as context plus detail and the cycle repeats.

Is there something theoretically novel here? If not, the RAG + LLM approach could be presented as a new implementation of an existing idea, though this would limit its scientific contribution. If there is a genuine theoretical innovation, it would be helpful to elaborate on new predictions that the RAG + LLM framework makes.

At present, the model appears to be primarily the former – a new implementation of an established procedure. Unfortunately, it is also a rather opaque implementation, given that we do not understand LLMs much better than we understand human cognition. Offering more concrete predictions would clarify the intended level of correspondence between the neocortex (modeled by the LLM) and the hippocampus (modeled by the RAG mechanism).

Apart from this there are several minor points which could be clarified:

1. A small-detail error is that in Fig 3i) the inverse frequency values on the x axis are 1000-times too low.

2. Why, in Figure 4 a, b, f), does the number of categories vary from panel to panel?

3. To evaluate their model, the authors attempt in 2.2.1 to compare the model's consolidated stories to a classical experiment of F.C. Bartlett, measuring cosine distance between an encoded and consolidated version over time as well as semantic intrusions.
- Unfortunately, this comparison stays rather fuzzy. What, for example, is measured in the Bartlett experiment plus its replication and how does it compare to the author's measures?
- Also, how does the analysis with different background data compare to Bartlett's experiment exactly with distortions in the direction to student's background knowledge. How does table S5, for instance compare with human data?
- A minor, additional, point is that this section refers to Figure 4 that appears only much later in the text, while many panels in Figure 3 were not yet addressed.

4. The comparison with Raykov et al. (2023) and HIPPOCORPUS data is more informative, but we have some concerns about transparency and reproducibility. Since it is unclear whether the model will remain publicly accessible, the results may be difficult to verify later. It might have been clearer to use more lucid NLP measures such as semantic diversity. Also, we could not find the exact GPT-4 scoring prompt in either the main text or the supplementary.

5. In Part 2 of the paper (Section 2.3), the authors use a smaller language model—GPT-2 Medium instead of Mistral – to test whether the RAG method enhances generalization. However, most of this section (and Figures 5 and 6) appears to focus on training the model from scratch on random graphs, which, as we understand it, is not the RAG method itself. This primarily demonstrates that GPT-2 can learn a graph based on spatial relations, but how does this differ from what Whittington et al. (2020) already showed using a simpler architecture? Moreover, if consolidation is not explicitly modeled – at least not in the originally proposed sense of a generative process involving RAG – then it remains unclear how this setup reflects the finding that "a shared abstract representation of different spatial environments with the same structure develops in the cortex through consolidation."

6. As we understand it, RAG is used only in Section 2.3.2 to learn a new task. However, the main outcome appears to be that RAG training aids in discovering the missing link, whereas the hippocampus model identifies it with less accuracy via random guessing from retrieved sequences that include the link? That said, we may be misinterpreting this point, as we are not entirely sure we understand the procedure illustrated in panel (i) correctly.

(Remarks on code availability)
No comment.

Reviewer #4

(Remarks to the Author)
I co-reviewed this manuscript with one of the reviewers who provided the listed reports. This is part of the Nature Communications initiative to facilitate training in peer review and to provide appropriate recognition for Early Career Researchers who co-review manuscripts.

(Remarks on code availability)

Reviewer #5

(Remarks to the Author)
Summary:

In this manuscript, the authors report on a set of numerical experiments involving large language models (LLMs). They use an xRAG model to simulate interactions between the hippocampus and the neocortex during learning. The central hypothesis is: hippocampus stores compressed representations of sequential memories, and neocortex selectively retrieves them into the working memory to perform sequence processing tasks. The neocortex, modeled by a LLM, can be trained on data replayed from the hippocampus, which can be interpreted as memory consolidation. Through memory reconstruction tasks, the authors use the model to reproduce various phenomena, including forgetting, changes in narrative attributes over time, and memory distortions due to prior knowledge. The simulation results qualitatively align with observations from human data. Finally, the authors use relational inference tasks to show that after consolidation, the model not only memorizes episodic memory, but also learn semantic knowledge from the shared structure of the input sequences.

Major issues and questions:

1. At the beginning of the manuscript, it would be helpful to have a clear statement of motivation describing what the authors are trying to achieve. The study is phrased as an attempt to understand memory consolidation, but do the authors want to understand memory consolation in the brain, or are they interested in LLMs? Are they hoping for mechanistic insights that would explain phenomena related to memory consolidation observed in humans, or are they merely trying to reproduce those phenomena? Given that the reported results mostly concern the output of the neural network (apart from section 2.3.1), one could perhaps characterize this work as a behavioral study on learning in which the subject happens to be an LLM. If this model is meant to explain the observed phenomena, more would have to be done understand how and why they come about by studying the internal representations of the network.

2. The model class under investigation is very complicated (not as complex as the brain, but sufficiently so that LLMs are essentially viewed as black boxes), and augmented by many idiosyncratic modeling choices made by the authors (see e.g. the next point). While is it certainly interesting that these models can reproduce certain behavioral observations from human memory consolidation, the complexity of the model class used here makes it difficult to asses whether the ability to reproduce these observations is surprising and/or robust. Some of the results appear to be rather generic consequences of learning in neural networks, while others are probably very dependent on the specific modeling choices made here. Can the authors provide some insight on which of their results are robust to model variations?

3. While it seems reasonable that the hippocampal representations are modeled as compressed, the particular setup of using a single (highly compressed) token as the gist and augmenting it by uncompressed details seems strange. This corresponds to first performing a very lossy compression, but eventually using many more tokens than necessary by adding raw details, which doesn't amount to an efficient compression scheme. Presumably compressing the whole episode into a few tokens (rather than one) would lose little information and be more efficient. What motivates this particular choice of input statistics, and why would this correspond to what the brain does?

4. The authors repeatedly emphasize the bidirectional interactions between hippocampus and cortex, but their model appears to be a feedforward network. (The authors declare that the inputs initiating memory retrieval should be considered as coming from cortex, but that seems to be the only influence from cortex on hippocampus). It should be clarified that what cortex (the LLM) does in the model doesn't affect the evolution of what is stored/retained in hippocampus. Is this by design? Do the authors believe that this is reflective of what happens in the brain?

5. Besides section 2.3, consolidation is only examined through the episodic memory reconstruction tasks. Other types of tasks are needed to show that semantic knowledge is learned and that neocortex can use it to answer questions beyond simply replaying the events. Similarly, it would be interesting to test the model on more general tasks before consolidation, to study neocortex's ability to selectively extract task-relevant information to guide output generation. This could establish whether the representation serves merely as an efficient compression, or also as a flexible format that supports downstream processing in the neocortex.

6. For the relational inference tasks in section 2.3, the model used is drastically different from the model used in previous sections. GPT-2 Medium (even though smaller than the previous model) is still quite large for simple relational inference tasks, and the idea of compression is not studied. Why? It's a bit unclear how this section fits into the larger picture of the manuscript. Was the idea simply to show that neural networks can learn structure in data ("semantic memory"), given that we're not sure how structured and generalizable the representations of the network were in the previous sections when it was processing language? How is this different from what has previously been done in the literature, e.g., in the Tolman-Eichenbaum-Machine paper?

7. The authors broadly connect their work to many parts of the literature, but what is missing is a concrete comparison of their model with other published models of memory consolidation. What specifically did we learn from this model compared to what was known before, and how do its predictions differ from those of other models? Putting these results in context by relating them to previous (often simpler, and more interpretable) models would be valuable here.

8. In terms of writing, the language used for reporting results appears to be strongly influenced by the interpretations the authors impose on their observations. (E.g. in section 2.2, how do we know that the information stored in the LLM corresponds to semantic memory derived from the episodic inputs?). This occasionally makes it difficult for the reader to extract objective descriptions of what was actually done and what was measured. With a more careful choice of wording, and a clearer distinction between methodology/observations and interpretation, this could be improved.

Minor issues and questions:

1. What does the text embedding model and the projector in the xRAG model correspond to in the brain? Are they really part of the neocortex?

2. More explanation is needed on the generation of surprising details. Some equations could be added in the supplementary section for explanation. Are "surprising details" a set of phrases or a sentence from the story?

3. For some events, the consolidated memory has a longer life span, while others have a shorter life span. Can this effect be reproduced in (and possibly predicted by) the model?

4. Fig3(b,c): Why is the original story more similar to the reconstruction than the encoded memory at the beginning of (b) and the end of (c)?

5. In the priors experiment, background stories and the Bartlett story are trained together. So the background stories aren't truly previous experiences. Do the results change if the training is done sequentially?

6. In the priors experiment (line 218), is the raw text used for fine-tuning? Or is the model fined-tuned on reconstructions of encoded stories? If it is fine-tuned on raw texts, why include the Bartlett story?

7. How would the batch size of consolidation affect forgetting? For example, for 100 memories to consolidate, does it matter if we take 2 memories per stage and 50 stages vs. 50 memories per stage and 2 stages?

8. Typo: For Fig5(e) should the correct output be "ye", rather than "o"?

9. Is the model in 2.3.2 fine-tuned from the model in 2.3.1? Or is it trained from scratch? Shouldn't the model from 2.3.1 be able to perform the task in 2.3.2 even without RAG?

10. Couldn't the distortion effect observed in Fig. 4a appear in any neural network that has learned to represent a certain input distribution and is then gradually trained on an out-of-sample input ?

11. What are the error bars in Fig. 3? Specifically, those in panel d) and g) look tiny. What is the statistical test being performed here?

(Remarks on code availability)

Reviewer #6

(Remarks to the Author)
I co-reviewed this manuscript with one of the reviewers who provided the listed reports. This is part of the Nature Communications initiative to facilitate training in peer review and to provide appropriate recognition for Early Career Researchers who co-review manuscripts.

(Remarks on code availability)

Version 1:

Reviewer comments:

Reviewer #1

(Remarks to the Author)
The authors seem to have made some nice improvements in response to other reviewer comments. I continue to be supportive of this work, without any further concerns.

(Remarks on code availability)

Reviewer #2

(Remarks to the Author)
The authors have significantly improved the clarity of the manuscript. From the perspective of neural activity data, I consider that the proposed model still exhibits some important discrepancies relative to neurobiological studies, e.g., memory replay. However, based on psychological data, the model proposes an interesting novel view of hippocampal-neocortical interplay during the encoding, consolidation, and retrieval of episodic memory. How these cortical regions interact at various stages of episodic memory processing has been an important topic in neuroscience, and the proposed view can enhance related future studies. This reviewer has no further comments on the manuscript.

(Remarks on code availability)

Reviewer #3

(Remarks to the Author)
The authors have made their best attempts at addressing all the points raised by the reviewers and the clarity of the paper has much improved, but in our view its fundamental "epistemological" weaknesses remain. Except for the very positive reviewer 1, these were noted by all reviewers and expressed in complementary form. Here, for the sake of brevity, we refer to the new Table 1 and to the slightly modified Fig. 1.

Table 1 lists, in the first column, three basic questions that, we all agree, have been around for decades. In the second column it lists, as "our" hypotheses, very reasonable general ideas that we appreciate are also "theirs", but are shared with many researchers in the field. Not necessarily by all: as to the first question, for example, we subscribe to the first generic hypothesis that "SM develops through the consolidation of EM", less to the more specific one that "general knowledge can be derived by prediction error minimization on specific memories" – although the "can" would make us agree that it is indeed a possibility in some particular instances, why not? Others, who see prediction error minimization everywhere, will probably embrace the statement wholeheartedly. Other statements in the column are likewise extremely hard to disagree with, such

as “consolidation mechanism produces ‘approximation of approximation’” or “EM and SM combine in working memory to solve problems”. The third column is not really (cognitive neuro)science but a summary description of an LLM implementation. Fine, nobody would want to argue with it and impose on the authors a different implementation. The fourth column spells out the achievements of the implementation, which are also fine but, if we may, nothing to write home about. LLMs are capable of astonishing feats (unfortunately!), so it is hard to be impressed that they can produce “learning in non-linguistic tasks”, “a similar pattern of memory distortions to human data” or “episodic contributions to problem solving”. More telling, for us, is the fifth column, which lists the paucity of relevant/competing models. They are not so few, in fact, but perhaps the right question is: when is it that a cognitive neuroscience model becomes something more than a concise formalized summary of a set of ideas, and develops into a tool producing an original contribution? This could be in formulating predictions not derivable from the ideas alone; or in enabling simulations that allow for quantitative falsifiability not attainable with qualitative reasoning; or a mathematical formalism with eg theorems and formulas; or in other forms. Maybe developing properly formalized models in this domain has been limited by the complexity of episodic and semantic memory and their interactions, which makes any reasonable prediction verifiable in some example, real quantification difficult, and mathematical theories too complicated.

So, we are left with the cartoons of Fig.1, which are fine, but they illustrate some cognitive neuroscience inspiration for LLMs, rather than LLM contributions to understanding human memory. Curiously, the cartoon that in the original submission was coming closer to serving the latter role, ie the right half of panel d), has been expunged in the revision, as if to stay clear of any definite neuroscience statement.

Other aspects of the response to the reviews are also not convincing. For example, we asked whether the LLM used for scoring will remain accessible, and the response “we have saved the scoring data in the GitHub repo to enable the raw data to be examined if the future model changes” does not really allay our concern, of what happens once the GPT4 used for scoring is not available any more. We would not want these other aspects, however, to take precedence before the critical “epistemological” worries.

(Remarks on code availability)

Reviewer #4

(Remarks to the Author)
I co-reviewed this manuscript with one of the reviewers who provided the listed reports. This is part of the Nature Communications initiative to facilitate training in peer review and to provide appropriate recognition for Early Career Researchers who co-review manuscripts.

(Remarks on code availability)

Reviewer #5

(Remarks to the Author)
We thank the authors for their detailed responses to our questions. Overall, the manuscript appears to be significantly improved. In particular, it is much clearer now what the authors wanted to achieve with this study, and we appreciate the additional results on problem solving. Motivating the paper in terms of the three hypotheses introduced at the beginning substantially improves the readability of the paper, even though these are of course not hypotheses that are being tested here — instead, the memory consolidation properties hypothesized by the authors are very much baked into the construction of the model.

Because of this, we tend to agree with one of other reviewers who noted that much of this manuscript appears to describe an engineering effort of putting together existing model components in a way that agrees with the authors' ideas about how memory consolidation in the brain should work. That is not necessarily a bad thing in our opinion, but the question remains: What did we actually learn about the brain from this study? Or in other words, are the models presented in this manuscript more than the sum of their parts, in the sense that they teach us something unexpected?

Given the mix-and-match, black-box modeling approach of this manuscript, it’s unclear whether we have any right to expect concrete answers to these questions, but we believe that the construction and characterization of these models nevertheless constitute an achievement that many readers will be interested in.

This review was conducted jointly with a junior researcher in our group. Below are some additional comments/questions from them:

1. In the rebuttal letter, the authors mention that surprising details they use are phrases but not sentences. However, in Table S1, the surprising details are shown as a sentence in each example. Also, Fig 3a shows that one detail is around 10 tokens, so the detail is most likely a sentence rather than one phrase.

2. In section 2.1.1 and section 4.2.1, the authors explain that they select phrases as surprising details by comparing perplexities on inputs of format: [gist token] + [tokens for phrase p]. If we understand it correctly, the perplexity measures the chance that the LLM generates phrase p right after the gist token. But a high perplexity doesn't necessarily mean that

information about p is missing from the gist token -- perhaps p is just supposed to be generated some tokens away from the gist token. The detail in example 1 of Table S1 still has some overlap with the content stored in the gist vector, so perhaps it would be better to rephrase surprising details as supporting details.

3. How accuracy is calculated in Fig 3d is a bit strange. Since the correct answer is known, why not directly use it to check the model's answer (perhaps use gpt-4o for automation)? Why additionally introduce two incorrect answers and then compare the distances in the embedding space? The accuracy by the authors' method could be heavily influenced by how incorrect answers are sampled.

4. Figure 5e in line 269 should be Figure 5f.

5. The experiments on content distortions (section 2.2.1) exclude the compressive retrieval process (use the original Bartlett story for consolidation), but the experiment on structure distortions (section 2.2.2) include it. Why such a difference? In section 2.2.1 the authors mention that they want to isolate the effect of consolidation from encoding, but the same could happen for experiments in section 2.2.2. Since it seems that a major point of this paper is compressive retrieval-augmented generation, I believe it would be better to use the full framework throughout the paper.

6. Similar to the previous point, experiments in section 2.3 don't involve compression of input sequences. To make the experiments more consistent throughout the paper, a potential improvement would be developing a custom xRAG model that compresses a sequence sampled from a spatial graph or family tree to a single token.

(Remarks on code availability)

Reviewer #6

(Remarks to the Author)
I co-reviewed this manuscript with one of the reviewers who provided the listed reports. This is part of the Nature Communications initiative to facilitate training in peer review and to provide appropriate recognition for Early Career Researchers who co-review manuscripts.

(Remarks on code availability)

Open Access This Peer Review File is licensed under a Creative Commons Attribution 4.0 International License, which permits use, sharing, adaptation, distribution and reproduction in any medium or format, as long as you give appropriate credit to the original author(s) and the source, provide a link to the Creative Commons license, and indicate if changes were made.
In cases where reviewers are anonymous, credit should be given to 'Anonymous Referee' and the source.
The images or other third party material in this Peer Review File are included in the article's Creative Commons license, unless indicated otherwise in a credit line to the material. If material is not included in the article's Creative Commons

license and your intended use is not permitted by statutory regulation or exceeds the permitted use, you will need to obtain permission directly from the copyright holder.
To view a copy of this license, visit https://creativecommons.org/licenses/by/4.0/

Many thanks to all the reviewers for their insightful feedback on our manuscript. We address the comments one-by-one below, but to summarise the major changes:

- We have made the paper's contributions, the predictions of the model, and its relation to other models clearer, e.g. by framing the paper around three key questions. (See the updated Introduction, and the new Table 1 and Figure 8.)
- We have added extra content to the Methods and Supplementary Information, both to clarify aspects of the modelling which were unclear, and to assume less prior knowledge of LLMs. We hope this will make the paper of interest to a broader range of readers.
- We have improved the modelling of narrative memory by testing 'semantic' memory through question answering, rather than just 'episodic' memorisation of the story, changing aspects of the simulation to better reflect the process of consolidation, and adding more experimental data for comparison.
- We have expanded the inference results, e.g. to demonstrate how inferences can be made by combining information from *multiple* episodic memories, and to visualise the representations in the family tree case. We have also tried to better explain how these simulations model the acquisition of semantic knowledge and hippocampo-neocortical interactions in problem solving more generally, so that they do not seem disjointed from the rest of the paper.
- We have improved the modelling of the hippocampus. Rather than merely sketching out a possible implementation of the hippocampus in the supplementary information, we have now modelled how a combination of autoassociative dynamics for pattern completion of a single item and heteroassociative dynamics for prediction of successive items could support episodic memory. Whilst not the focus of this paper, this illustrates the hypothesis that hippocampus and neocortex use very different mechanisms for sequence memory. (See the updated Methods and Figures 9 and S1.)

Reviewers two, three, and five requested a clearer explanation of the novel contributions of the paper. We summarise them as follows:

- This paper explores three main questions (see Table 1):
    - *How does episodic memory influence semantic memory?* We suggest that semantic memory develops through the consolidation of episodic memory, with general knowledge derived by prediction error minimisation on specific memories. We implement this in the model as a consolidation mechanism: sequences in the model hippocampus are used to train the model neocortex.
    - *How does semantic memory influence episodic memory?* We suggest that episodic memory is compressed based on semantic memory, with a minimal subset of information stored for approximate reconstruction of an event. Episodic memories bind together content at different levels of abstraction, e.g. a schema for a meal at a restaurant and the flavour

of a particular dish, which neocortex then 'decompresses' during recall. As replayed memories are themselves a reconstruction, consolidation produces an 'approximation of an approximation'. We implement this in the model as a compression mechanism, making use of 'context compression' techniques from the LLM literature: each episodic memory is stored as a vector representing the gist, plus residual poorly predicted elements, from which the LLM can approximately reconstruct the sequence via RAG.

  - *How do semantic and episodic memory interact?* We suggest that episodic memory and semantic memory combine in working memory to recall events and solve problems; episodic memory is retrieved from the hippocampus, while semantic memory makes inferences about the past and predictions about the future based on this. We implement this in the model as retrieval-augmented generation: episodic memory is the 'in-context' sequences retrieved associatively from hippocampus, and semantic memory is the 'in-weights' information in the generative network.

- By simulating the encoding, consolidation, and recall of narrative stimuli, we show that the model predicts human data on changes to memories over time, and captures gist-based distortions seen in human memory. By simulating human behaviour in relational inference tasks, we show how problem solving is supported by the mapping of new stimuli to a learned abstract representation in semantic memory, and show how this can be supported by retrieval of relevant episodic memories from the hippocampus.
- As described above, we propose a computational model in which the hippocampus stores events and then replays them to teach a deep generative model in the neocortex to predict the next element in each sequence. We simulate this neocortical network using an LLM. The LLM learns the essence of specific episodes, but also statistical regularities that transfer to new tasks. Several papers consider memory as a predictive process relying on generative models (Hemmer & Steyvers, 2009; Nagy & Torok, 2020; Nagy et al., 2025), but fewer consider consolidation as a mechanism for training generative models on replayed memories (Fayyaz et al., 2022; Spens & Burgess, 2024) and to our knowledge no existing work models the *consolidation of sequential events into a generative model* specifically. Most importantly, no other work models how episodic and semantic memory work together during encoding, retrieval and problem solving.
- Whilst many papers explore the behaviour of pre-trained LLMs on cognitive tasks, we have not come across any papers that model human *memory* for 'naturalistic' linguistic stimuli using LLMs (except the modelling of event segmentation in Michelmann et al., 2025), although some recent work takes inspiration from human memory to improve LLMs (e.g. Fountas et al., 2024). The spatial and relational inference tasks could be modelled with alternative

approaches too, but it is interesting that the same kind of model can learn all of these things through prediction error minimisation on replayed memories, unifying different functions.

- Crucially, we hope our model of how episodic and semantic systems cooperate in working memory is novel, contributing recent specifics (retrieved from the hippocampus) and generalities (predicted by the neocortex, given the hippocampal 'context') respectively. Whilst this possibility is discussed in Dong et al. (2025), to the best of our knowledge this is the first work to model this. Furthermore, we show how hippocampal traces could combine gist and detail, reducing the 'size' of memory traces in hippocampus, and enabling efficient reconstruction. Whilst it is known that memories in hippocampus are abstracted and compressed (Quiroga et al., 2012), there is little computational modelling of how this compression occurs for text and other 'naturalistic' data. (Note that the combination of RAG with consolidation and compression mechanisms is new, and makes different predictions from RAG alone; see Figure 8.)
- In summary, we hope the model suggests a novel way of thinking of the roles of hippocampus and neocortex in memory processing as an adaptive, compressive form of retrieval-augmented generation, in which: i) the hippocampus and neocortex play complementary roles in problem solving, contributing recent specifics and generalities respectively, ii) the hippocampus trains the neocortex by replaying its memories during consolidation, and iii) the neocortex compresses memories before they are stored in the hippocampus.

Reviewer #1:

This paper uses GPTs as models of hippocampal-cortical interactions during consolidation and retrieval. The simulations demonstrate how prior knowledge can bias memory recall, how information can be generalized over the course of consolidation, and how hippocampal memory retrieval can provide useful context for cortical prediction. This is, to my knowledge, the first systematic attempt to map components / functions of GPTs to memory systems and their interactions. There are several interesting insights, and I think the paper will make a highly valuable contribution to the literature. I expect many others will be interested in adapting and building on this framework.

I reviewed a version of this paper at a prior journal and was already very positive about it then. This version has focused on a subset of the simulations and added some depth to these. My original suggestions have already been incorporated and this version does not raise any new concerns for me.

Thank you for your positive feedback!

We're glad you think 'many others will be interested in adapting and building on this framework', as we intend to release a Python library to enable this upon publication of the paper (in addition to the code to reproduce the results).

Reviewer #2:

This paper proposes a computational model explaining how the hippocampus (which stores episodic memories) and the neocortex (which stores general knowledge or semantic memory) work together. They model this interaction using ideas borrowed from machine learning, specifically retrieval-augmented generation (RAG) — a technique used in large language models (LLMs) like Chat GPT. More precisely, the model hypothesizes: 1) the neocortex acts as a generative model trained to predict experiences like an LLM for text processing; 2. the hippocampus stores episodic memories in a compressed form consisting of gist sequence plus surprising details; 3. the neocortex learns from hippocampal replays, extracting general patterns (semantic memory); 4. the hippocampus retrieves relevant memories, which in turn guide the neocortical model to generate coherent responses, like retrieval-augmented generation (RAG) in AI systems. The authors compared the model's performance with that of simpler models in AI as well as humans in spatial and narrative memory tasks. Based on these results, the authors suggest that the hippocampal-neocortical network functions similarly to AI foundation models that learn through prediction error minimization.

I reviewed the manuscript, assisted by a postdoc in my lab. We found that some results are interesting but also have several concerns about the manuscript. In short, we feel the manuscript is not clear enough and confusing, although neither of us is an expert in AI research, and we might have missed some essential points about the results. Below, we list our comments.

Thanks very much for your feedback. We are glad you found some results interesting, and hope that the changes described below clarify the confusing aspects of the paper.

Major comments:

1. We were first excited to read the manuscript, but our enthusiasm dropped rapidly as we proceeded. The model uses two successful models in modern machine learning, Chat-GPT2 or Mistral 7B for the neocortex, which was trained on sequences representing hippocampal replay activities. The trained model exhibited impressive performance in various memory tasks. However, in a sense, the model's high performance is guaranteed from the beginning, as we already know the component models perform their own businesses excellently. However, the combined model will not be the best, as the component models are not the state-of-the-art models. Therefore, from machine learning point of view, the manuscript does

not seem to show sufficiently novel results. (However, if this study does show novel results in ML, the results should be explained.) On the other hand, the model is not strongly grounded in biological neural networks of the hippocampus and prefrontal cortex, although it explains some psychological phenomena observed in episodic memory. The authors are aware of the lack of neural-level support for the model (as they stated in the discussion), but we are still left with a doubt. After all, what novel points have we learned from this model about the hippocampal-neocortical mechanism of memory processing?

The purpose of the model is not to improve on baselines in the ML literature, but to shed light on how memory works in the brain. We hope the summary of the contributions of the paper at the start of this document helps to clarify what we learn from the modelling.

As mentioned above, we have reframed the paper in terms of three main questions the modelling seeks to address (Table 1). We have also clarified how the predictions of the model differ from those of alternatives (Figure 8).

2. As many readers in neuroscience will only poorly know the machine learning models, especially Mistral 7B, the authors should explain the structure and function of these models in somewhat detail. In addition, we wonder whether the authors actually modeled the hippocampus or merely used gist sequences and surprising details (generated by the neocortex?) as its replay activity.

Thanks for suggesting this – you're right that the previous version assumed too much prior knowledge. We have added a 'Large language models' section at the start of the Methods, as well as more clearly pointing readers towards existing content in the Supplementary Information.

Regarding the hippocampus: We originally used gist vectors and surprising details directly, and whilst we sketched out a compatible approach in the supplementary information, we did not confirm it could memorise the stimuli sequences well.

We have now modelled the hippocampal formation more fully. To motivate this, recall requires both pattern completion of the current item and prediction of the next one. For example, recalling a day out from a photo requires first retrieving the scene depicted in the photo, and then remembering the events following and preceding that scene. Modelling sequence memory therefore requires a mechanism that supports both autoassociative reconstruction of the present state from a partial input and heteroassociative transitions to future states (Sompolinsky & Kanter, 1986).

Here this is implemented with a pair of modern Hopfield networks. A standard modern Hopfield network (MHN) retrieves items from their noisy versions, while an asymmetric MHN (Chaudhry et al, 2023) retrieves the next item for a given item. This simple model demonstrates the idea of combining autoassociative and heteroassociative connectivity in a 'biologically plausible' way, but many alternative

implementations of sequence memory exist, e.g. (Karuvally et al., 2025), and could be compatible with this proposal. In the narrative simulations, the xRAG gist vector for an event can be retrieved autoassociatively from a cue, while each token in the sequence of details can be retrieved heteroassociatively from the previous token.

(Having confirmed that the model hippocampus could recapitulate sequences reliably, it didn't add much to use the MHNs, so to avoid unnecessary computational cost we used the memory sequences directly in some simulations.)

3. If we understand correctly, the most essential hypothesis of the model is that the hippocampal-neocortical neural system works as retrieval-augmented-generation (RAG) proposed in ref. 31. Is the present model just a biological implementation of the original model? Alternatively, does it offer a novel function that was missed in the original model? The author should argue for experimental evidence supporting this assumption, if any.

The compression and consolidation aspects we combine with RAG are novel. Combining these different components into a single system that makes behavioural predictions is the intended contribution.

We have clarified where the full model - RAG plus consolidation and compression mechanisms - makes different predictions from the constituent parts in isolation (Figure 8). Without the compression element, the model cannot explain distortions in newly encoded memories, and without the consolidation element, the model cannot explain increasing similarity to priors.

4. The model is meant to give insight into hippocampal-neocortical interactions, however, it is hard to understand the actual role of the hippocampus in the model. Figure 1a) shows State n and State n+1 with some illustration, which is not explained in the caption. Similarly, what the States here represent, and why only n and n+1 is included.

We have now modelled the hippocampal formation more fully, rather than just sketching out a compatible model in the supplementary information (see above). We have added Figure 9 to show the difference between the autoassociative mechanism by which a state is retrieved from a partial or noisy version, and the heteroassociative mechanism by which the next state is retrieved for a given state.

The diagram shows state n and n+1 because sequences are retrieved by iteratively retrieving the next state, i.e. only one-step transitions are stored. (This is common for models of sequence memory.) We have amended the caption for Figure 1a to clarify that the hippocampus is modelled with modern Hopfield networks (Ramsauer et al., 2020) storing states and one-step transitions between them. (As noted above, having confirmed that the model hippocampus could recapitulate sequences reliably, it didn't add much to use the MHNs, so to avoid unnecessary computational cost we used the memory sequences directly in some simulations.)

We have updated the Methods to clarify these points.

5. Figure 1, which should provide a clear overview of the model, looks very dense and becomes difficult to understand. Some things remain unexplained. If these things are not necessary, we would recommend leaving them out of the Figure to avoid confusion. Alternatively, they should be explained in the caption. For example, explanation for 1c) only states "The neocortical and hippocampal networks can work together to recall memories and solve problems via RAG.", but the meaning "New task" is unclear. The meaning of the color scheme is also not clear.

As it was difficult to remove panels without losing key information, we have improved the caption, adjusted some of the wording in the figure (e.g. removing the 'retriever' and 'generator' terminology), and made other minor improvements. We also hope the newly added Table 1, which now precedes the figure in question, addresses some of the confusion.

6. According to Fig. 1b, the hippocampus stores sequences in a compressed form in gist sequences and surprise details of specific episodes. Table 1 shows examples of the stories retrieved from the gist and detail of the hippocampal representations. However, the gist-only stories are more complex than the original ones in these examples, and they do not clarify how stories are decomposed into gist and detail by the hippocampus. Some examples of explicit hippocampal representations will be useful for readers unfamiliar with LLMs, although I am unsure whether showing such representations is possible.

In Table 2 (previously Table 1), the 'Recalled text' column does not correspond to stored hippocampal representations. In the row for the 'Encoded' story, only the gist (xRAG) vector is stored in the model hippocampus, and 'Recalled text' is the output of the system. So the fact that the recalled story features semantic intrusions does not reflect a more complex hippocampal trace – these intrusions are added by the model neocortex during recall.

See additions to the Methods and the newly added Figures 9 and S1 for more information about the model hippocampus. In particular, Figure 9 contains a schematic showing the hippocampal representations, which we hope addresses the point above.

We have also updated the Methods to better describe how the unexpected details are identified.

7. In Fig. 3a, the authors showed a trade-off between efficiency and accuracy of memory recall. How do the hippocampus and neocortex differently contribute to efficiency and accuracy? Which component is more critical to the trade-off? What does "simply imagining the event" mean?

Figure 3a shows several options for the initial encoding of hippocampal memory. In the ‘Full’ case, the original text is stored in full, so the memory size is at a maximum (i.e. the storage efficiency is poor). When recalled using RAG, accuracy is high because the original text is retrieved from the hippocampus to prompt the neocortex. At the other end of the spectrum, no data is encoded in the hippocampus in the ‘Imagined’ case, i.e. ‘simply imagining’ the event means using the LLM alone without any retrieval of a hippocampal trace. As this is prior to any consolidation, the LLM’s output is just its ‘best guess’ at the story’s continuation, hence accuracy / similarity is poor. The three bars in the middle all use the hypothesised gist plus details encoding, but with different numbers of details. The more details are stored in hippocampus, the more is retrieved to inform the neocortical predictions during RAG, and thus the better the accuracy. The bars are arranged on the x axis in order of the amount of data in hippocampus, ranging from no data in the ‘Imagined’ case to every detail in the ‘Full’ case. The amount of data encoded in hippocampus affects the accuracy of recall, i.e. there is a trade-off between these objectives.

8. In Fig. 3b and c, the meaning of Original and Encoded is not explained. Is similarity the cosine similarity in the embedding space? This should be stated somewhere. Similarity of both “Encoded” and “Original” is increasing. Which one is the neocortex’s reconstruction?

Sorry that these plots were unclear.

Figure 3b and c show the cosine similarity in embedding space of the neocortical memory (i.e. the LLM’s continuation of the start of the story) compared to i) the ‘Original’ story and ii) to the version that is actually replayed, i.e. the story reconstructed from what was ‘Encoded’.

To clarify, the ‘Original’ story is encoded in compressed form. During consolidation, the reconstruction of this compressed memory is replayed to train neocortex, and this is denoted as ‘Encoded’.

We have clarified this in the caption.

9. In Fig. 4g and 4h, the authors compared narrative distortion in memory recall between humans and the model. For short delays, both agents exhibited similar tendencies in making omission and extension errors. In contrast, for long delays, the two error types display largely different proportions in humans but similar proportions in the model. Please explain why so, if possible.

The short delay corresponds to retrieval-augmented generation, whereas the long delay corresponds to the consolidated memory (i.e. is reliant on the LLM alone). One possible reason for your observation is that LLMs such as Mistral tend to be overly verbose compared to humans, leading to more extension and fewer omission errors overall, but the RAG setting reduces this tendency (as the model is encouraged in its training to only use the provided information). However hopefully the comparison

between the updated and incomplete stories is still meaningful.

10. Spatial navigation is the most extensively studied subject in the research of the rodent hippocampus. Figure 6d presents the results of PCA of the representations extracted from different environments, and the authors demonstrated that the model outperforms pre-trained GPT-2 without a neocortex circuit. However, since the spatial environments studied here are simple, it remains unclear to what extent the results confirm the model's excellent performance in spatial navigation. For instance, can the model learn a shortcut from sample trajectories that do not involve it? What are gist sequences and surprise details in spatial navigation?

Here all the testing measures whether the model learns a shortcut from sample trajectories that do not involve it. The model is tested on sequences from new environments not used in training, so solving these tasks requires genuine inference, not just memory.

Some refinements to the inference simulations increased performance of both models (e.g. training a custom 'tokeniser' so that each location / family member was represented by a single token, regardless of the frequency of a pair 'xy' in the English language). This makes the evidence for generalisation abilities more compelling, e.g. see Figure 6h. We also added a visualisation of the family tree model's representations to Figure 7.

For simplicity the compression aspect is not simulated in the inference simulations, as it is already fairly complex. But xRAG could in principle represent a sequence as a compressed vector and sub-sequences that were poorly predicted, just as in the text simulations.

11. Related to comment 10, the baseline model (pretrained GPT-2) does not produce a topographic map of the environment, while "our model" does (Fig. 6a-d). Please explain the reason for this difference. Intuitively, Chat-GPT or Transformer-based LLM is thought to learn relationships between inputs based on their similarities. Therefore, I do not understand why GPT-2 alone cannot learn similarity between spatial locations.

The baseline model has not been fine-tuned on the task, and is unable to perform well even when given a lengthy spatial sequence. Its token representations reflect this. In contrast, GPT-2 trained on spatial sequences, simulating the consolidation of replayed episodic memories into the model neocortex, learns a common underlying structure: a 2D map. The token representations for an arbitrary item come to represent its inferred position in this map, because this is the most useful representation to predict the next observation in the sequence. (GPT-2 is only a small model, so it is possible that a larger LLM could infer that the sequences are spatial purely in context.)

Some refinements to the method in Figure 7g reveal that distances between token representations are somewhat correlated with Manhattan distances at early layers of the baseline model, presumably because early layers aggregate information from nearby tokens, and nearby locations are nearby in the sequence on average.

12. Indeed, section 2.3 and Figure 6 are quite confusing. Please explain what "our model" refers to in Fig. 6.

Our model refers to the GPT-2 architecture trained from scratch on spatial sequences, simulating the consolidation of replayed episodic memories into the model neocortex. We have tried to clarify this.

Minor comments:

13. Some panels, for instance, those in Figs. 3 and 4 are referenced in a random order in the main text. Although I do not think this is unacceptable, it decreases the clarity of the manuscript.

Thanks for spotting this – we have adjusted the ordering in which figures are referenced in a couple of places. (However in some places we have prioritised arranging subplots in the figure in a logical way even if this does not quite match the order in the text.) Throughout the paper, we have tried to improve the clarity of the figures.

14. It would be helpful to use colors consistently. For example in Figure 3, orange represents Memory size (a), "Encoded" (b and c), "Consolidated" (d), "Richness in detail" (e), "retold" (vs recalled) (g), Pre-consolidation word distribution (i). This makes it more difficult to quickly orient in the figures.

We have tried to adjust the colour scheme to make this figure clearer.

Thanks again for all your helpful comments, which we hope have improved the paper.

Reviewer #3:

This manuscript starts off from a very reasonable and widely shared perspective on the involvement of the hippocampus and neocortex in memory formation, retrieval/regeneration and consolidation, and develops it using machine learning and in particular large learning models. Importantly, in such a perspective episodic memories are not retrieved in the same exact form they were originally stored but are, instead, the result of a generative process which reflects schemas previously consolidated in the neocortex. This "semanticization" accrues progressively, leading to increasing deviations, also in the concrete example offered in the manuscript of short 3-4 sentence "stories", from the original.

Specifically, the authors consider that memory consolidation can be modeled as a generative network with Retrieval-Augmented Generation (RAG). In this framework, the neocortex is represented as a pretrained large language model (Mistral 7B at first, later GPT-2 Medium), while the hippocampus functions both as a prompter and as a retrieval database that encodes context, the gist and surprising detail of an episode. The interaction between the two brain regions is thus assimilated to a Machine Learning method (RAG) that augments any given prompt to the LLM with relevant context from the prompter/database.

Applying RAG iteratively is taken to model memory consolidation: the neocortical "generator" is gradually trained on context-augmented prompts from the hippocampal "retriever".

Prompts take the form of:
[INST] Refer to the background document and answer the question.
Background: {XRAG TOKEN}
Question: {FIRST LINE} What happened (in detail)?
Other details to include: {SURPRISING DETAILS}. [/INST] The answer is:

This is all fine and good. Except that the development of the general perspective which is offered in the manuscript is not science, in our view, but rather an engineering demonstration. Assertions and hypotheses – which by and large we would happily subscribe to – are not mathematically proven or empirically tested and validated but rather exemplified with a reduced scale toy model and put forward as cartoon figures. It is in fact not entirely clear what is reduced in scale, here, since no actual neural circuit is simulated, although probably the 500 episodic strings can be regarded as a reduced scale of the experiences concurrently held in memory by a human being (we write “human” because of the focus in the manuscript on language and linguistic episodes).

We agree there is a risk of presenting this work as engineering rather than science if we focus too much on the implementation as opposed to the hypotheses. To address this, we have tried to frame the paper around three scientific questions, as described at the start of this document, and state our hypotheses clearly.

We also hope updates to the simulations described below improve your assessment of whether the hypotheses are ‘empirically tested’.

A major concern for us is also that the authors could have more clearly articulated the conceptual logic of the RAG-inspired consolidation model, independent of the machine learning implementation. This would separate neuroscience talk from machine learning talk and could help highlight what is theoretically novel and what is not. It could perhaps be summarized as follows:

1. A sequential input from working memory (a story) is compressed as gist plus detail, in the hippocampus.

2. The first few sequences from working memory are presented to the neocortex which predicts the following sequences.

3. The neocortex then minimizes the prediction error between the compressed sequential input and its prediction and updates its weights.

4. Now the prediction is compressed as context plus detail and the cycle repeats.

Is there something theoretically novel here? If not, the RAG + LLM approach could be presented as a new implementation of an existing idea, though this would limit its scientific contribution. If there is a genuine theoretical innovation, it would be helpful to elaborate on new predictions that the RAG + LLM framework makes.

We agree we need to more clearly separate the hypotheses from the implementation to make the theoretical novelty of this work clearer.

As described at the start of this document, we believe there are several novel contributions. Most importantly, no other work models how episodic and semantic memory work together during encoding, retrieval and problem solving. Whilst there are many relevant prior models, we aim to address the three questions in Table 1 in a unified way, and existing models address only a subset of these. Furthermore, the combination of the 'RAG + LLM' framework with compression and consolidation mechanisms is novel. In addition, very little other work models human memory for narrative stimuli computationally.

We have tried to clarify these points in updates to the Introduction and Discussion, and with the addition of Table 1.

At present, the model appears to be primarily the former – a new implementation of an established procedure. Unfortunately, it is also a rather opaque implementation, given that we do not understand LLMs much better than we understand human cognition. Offering more concrete predictions would clarify the intended level of correspondence between the neocortex (modeled by the LLM) and the hippocampus (modeled by the RAG mechanism).

We agree that the current mechanistic understanding of LLMs is limited, but there is a trade-off between the simplicity of the model and the ability to apply it to naturalistic stimuli. Language is obviously central to memory for humans, and we hope that being able to model linguistic stimuli justifies the 'black box' nature of the model. This is an important point that we have added to the discussion.

Regarding more concrete predictions, we have added Figure 8 to summarise how the model's predictions differ from various alternatives.

Apart from this there are several minor points which could be clarified:

1. A small-detail error is that in Fig 3i) the inverse frequency values on the x axis are 1000-times too low.

Thanks for spotting this - we have fixed it as well as making other improvements to this figure.

2. Why, in Figure 4 a, b, f), does the number of categories vary from panel to panel?

Thanks for spotting the discrepancy between these parts of Figure 5 (previously, Figure 4) - the addition of the technology category in the PCA plot was a mistake (due to data cached from a previous run). In part f, we have added the extra word cloud for completeness.

3. To evaluate their model, the authors attempt in 2.2.1 to compare the model's consolidated stories to a classical experiment of F.C. Bartlett, measuring cosine distance between an encoded and consolidated version over time as well as semantic intrusions.
- Unfortunately, this comparison stays rather fuzzy. What, for example, is measured in the Bartlett experiment plus its replication and how does it compare to the author's measures?
- Also, how does the analysis with different background data compare to Bartlett's experiment exactly with distortions in the direction to student's background knowledge. How does table S5, for instance compare with human data?
- A minor, additional, point is that this section refers to Figure 4 that appears only much later in the text, while many panels in Figure 3 were not yet addressed.

We agree that our results in this section would be stronger with more quantitative comparison to experimental data.

In Bartlett (1932), participants either repeatedly retold the story at different delays (repeated reproduction), or communicated it from person to person (serial reproduction). Whilst this is the original reference, its descriptions of the repeated reproduction findings are very qualitative, so the Bergman and Roediger (1999) replication is a more useful source of data.

We've added Figure 5b to summarise data from this study. Participants read The War of the Ghosts and recalled the story after 15 minutes, 1 week, and 6 months; the plot shows the experimental subset (n = 8) who completed all three recalls. The story was divided into 42 propositions, and each recalled proposition was classified as accurate (no distortion), minor distortion (changes to the wording but not the

meaning), or major distortion (meaning-altering changes such as normalisation, inference, or importation).

Here normalisation refers to substitutions that make the recalled content more schema congruent. Unusual or culturally unfamiliar elements are replaced with more typical ones (e.g. replacing “canoe” with “boat”). Inference means that plausible details are added that were implied by the story but never explicitly stated (e.g. recalling that someone was struck by an *arrow* when the story only says they were struck). Importation means that new elements are introduced that have no clear basis in the original story. These are borrowed from other parts of the narrative or from general knowledge.

For each delay, the plotted values show the fraction of recalled propositions falling into each category, such that the three bars sum to one and omitted propositions are excluded. Note that intrusions that do not qualify as major distortions are also omitted, i.e. the term major distortions only covers distortions due to ‘normalisation, inference, or importation’. The ‘major distortions’ category is therefore a measure of semantic influence on episodic recall. This shows that more and more of the recalled story is distorted over time.

Whilst not identical, the similarity to the average of the topic is another measure of semantic influence on episodic recall, so our results agree broadly with the quantitative data from Bergman and Roediger (1999). The experimental data only involves one set of priors (those of the university student population the participants were gathered from), but we hope varying the priors makes the claim about the model more convincing.

4. The comparison with Raykov et al. (2023) and HIPPOCORPUS data is more informative, but we have some concerns about transparency and reproducibility. Since it is unclear whether the model will remain publicly accessible, the results may be difficult to verify later. It might have been clearer to use more lucid NLP measures such as semantic diversity. Also, we could not find the exact GPT-4 scoring prompt in either the main text or the supplementary.

Regarding whether the LLM used for scoring will remain accessible, we have saved the scoring data in the GitHub repo to enable the raw data to be examined if the future model changes.

The prompt is in the Methods, but we now refer to it more clearly in the main text.

5. In Part 2 of the paper (Section 2.3), the authors use a smaller language model—GPT-2 Medium instead of Mistral – to test whether the RAG method enhances generalization. However, most of this section (and Figures 5 and 6) appears to focus on training the model from scratch on random graphs, which, as we understand it, is not the RAG method itself. This primarily demonstrates that GPT-2 can learn a graph based on spatial relations, but how does this differ from what Whittington et al.

(2020) already showed using a simpler architecture? Moreover, if consolidation is not explicitly modeled – at least not in the originally proposed sense of a generative process involving RAG – then it remains unclear how this setup reflects the finding that “a shared abstract representation of different spatial environments with the same structure develops in the cortex through consolidation.”

The purpose of the inference simulations is as follows.

The narrative simulations use models with a lot of ‘general knowledge’ in their pre-trained weights, and do not fully address how this is acquired. Here we investigate more mechanistically how consolidation develops generalisable semantic knowledge.

Whittington et al. (2020) hard-code the distinction between structure and content, and use an architecture which is specific to relational inference. Here the model receives nothing but sequences of observations, with no priors about their meaning built into the network. The model can abstract the underlying structure (a 2D gridworld or a family tree) based on only prediction error minimisation.

These results also help to demonstrate that the same model can apply to linguistic and non-linguistic stimuli. The simpler task (as well as the simpler model) enables more analysis of the network’s representations.

The results in Section 2.3.1 show the model can do well at relational inference on a new 2D gridworld or family tree, and suggest a mechanism underlying this (now visualised for the family tree case too). We then extend this to consider inference from episodic memories, rather than inference from perception, which depends on the retrieval of relevant episodic memories into ‘working memory’ (the LLM’s context) through RAG.

6. As we understand it, RAG is used only in Section 2.3.2 to learn a new task. However, the main outcome appears to be that RAG training aids in discovering the missing link, whereas the hippocampus model identifies it with less accuracy via random guessing from retrieved sequences that include the link? That said, we may be misinterpreting this point, as we are not entirely sure we understand the procedure illustrated in panel (i) correctly.

Sorry if this section was written in a confusing way.

In the simulations above, inference was tested as the continuation of a single new (long) sequence in working memory. However working memory is limited, so the brain must *selectively* recall *relevant* experiences. In addition, problem solving can synthesise information from more than one episodic memory retrieved from hippocampus; multiple pieces of information encoded at different times can be recombined with semantic knowledge, e.g. when inferring that A is the sibling of C from the observation on one day that A is the child of B, and on another that B is the parent of C.

The point of Figure 6j and k is simply to show that neither the hippocampal model nor the neocortical model in isolation can explain problem solving from recent episodic memories, and to demonstrate that the combination can combine information from *multiple* memories to draw inferences that are impossible from individual memories. We have tried to clarify the procedure in the text, but in essence it is 'standard' RAG.

Thanks again for all your helpful comments, which we hope have improved the paper.

Reviewer #5:

Summary:

In this manuscript, the authors report on a set of numerical experiments involving large language models (LLMs). They use an xRAG model to simulate interactions between the hippocampus and the neocortex during learning. The central hypothesis is: hippocampus stores compressed representations of sequential memories, and neocortex selectively retrieves them into the working memory to perform sequence processing tasks. The neocortex, modeled by a LLM, can be trained on data replayed from the hippocampus, which can be interpreted as memory consolidation. Through memory reconstruction tasks, the authors use the model to reproduce various phenomena, including forgetting, changes in narrative attributes over time, and memory distortions due to prior knowledge. The simulation results qualitatively align with observations from human data. Finally, the authors use relational inference tasks to show that after consolidation, the model not only memorizes episodic memory, but also learn semantic knowledge from the shared structure of the input sequences.

Major issues and questions:

1. At the beginning of the manuscript, it would be helpful to have a clear statement of motivation describing what the authors are trying to achieve. The study is phrased as an attempt to understand memory consolidation, but do the authors want to understand memory consolation in the brain, or are they interested in LLMs? Are they hoping for mechanistic insights that would explain phenomena related to memory consolidation observed in humans, or are they merely trying to reproduce those phenomena? Given that the reported results mostly concern the output of the neural network (apart from section 2.3.1), one could perhaps characterize this work as a behavioral study on learning in which the subject happens to be an LLM. If this model is meant to explain the observed phenomena, more would have to be done understand how and why they come about by studying the internal representations of the network.

Thanks very much for your feedback.

We are chiefly interested in understanding memory in the brain, not LLMs. We agree there is a risk of presenting this work as engineering rather than science if we focus too much on the implementation as opposed to the hypotheses.

To address this, we have tried to reframe the paper around three scientific questions, as described at the start of this document, and clearly separate our hypotheses from their implementation. Please see also the newly added Table 1.

2. The model class under investigation is very complicated (not as complex as the brain, but sufficiently so that LLMs are essentially viewed as black boxes), and augmented by many idiosyncratic modeling choices made by the authors (see e.g. the next point). While is it certainly interesting that these models can reproduce certain behavioral observations from human memory consolidation, the complexity of the model class used here makes it difficult to assess whether the ability to reproduce these observations is surprising and/or robust. Some of the results appear to be rather generic consequences of learning in neural networks, while others are probably very dependent on the specific modeling choices made here. Can the authors provide some insight on which of their results are robust to model variations?

We acknowledge that certain modelling choices are 'idiosyncratic' or arbitrary, especially in terms of the method used for context compression. (This is a newly emerging field of research in the LLM literature so options for implementing some of our hypotheses were limited.) We agree that it is important to differentiate between what is central to the conceptual model vs. what might be consequences from one potential implementation.

As mentioned above, we have tried to start with three core questions, and state our hypotheses for each before diving into the computational model. For instance, consider the second of these questions: *How does semantic memory influence episodic memory?* We hypothesise that episodic memory is compressed based on semantic memory, with a minimal subset of information stored for approximate reconstruction of an event. What is consolidated is thus already an approximation. We implement this in the model by making use of 'context compression' techniques from the LLM literature: each episodic memory is stored as a conceptual gist, plus residual details not captured by the gist, from which the LLM can approximately reconstruct the sequence via RAG. However, this is where the modelling choices become more arbitrary: there are multiple other context compression approaches that could be used here in place of xRAG, e.g. ICAE (Ge et al. 2023), which compresses text to n memory slots where n is some fraction of the number of input tokens. The choice of xRAG mostly reflects implementation details (e.g. ICAE cannot compress very short texts). We have tried to make this distinction clearer throughout.

Regarding robustness to the choice of LLM, a previous version of this work used GPT-2 Medium for the text simulations (which was replaced with Mistral 7B to enable

the context compression experiments), and the results were broadly similar. Because of the fact that xRAG weights exist only for Mistral 7B , whereas GPT-2 is small enough to easily explore training from scratch, it is challenging to do a systematic comparison across LLMs.

However, the overall behaviour of the model and its predictions (e.g. Figure 8) are independent of the specific choice of implementation of compression or LLM.

3. While it seems reasonable that the hippocampal representations are modeled as compressed, the particular setup of using a single (highly compressed) token as the gist and augmenting it by uncompressed details seems strange. This corresponds to first performing a very lossy compression, but eventually using many more tokens than necessary by adding raw details, which doesn't amount to an efficient compression scheme. Presumably compressing the whole episode into a few tokens (rather than one) would lose little information and be more efficient. What motivates this particular choice of input statistics, and why would this correspond to what the brain does?

Thanks for pointing out this drawback of xRAG. We agree that compressing texts to a single token then using many tokens to encode details is odd, as the core of the memory takes up a fraction of the capacity of the more extraneous details.

As mentioned above, context compression is an immature field of research, so the options for modelling it are fairly limited. Furthermore the specific choice of xRAG is an implementation detail that is not central to the hypothesis. If even this crude method of context compression produces interesting semantic influences on episodic memory, more sophisticated approaches are certainly worth exploring in the future.

To address this comment, we have added a discussion of different context compression options to the Supplementary Information.

4. The authors repeatedly emphasize the bidirectional interactions between hippocampus and cortex, but their model appears to be a feedforward network. (The authors declare that the inputs initiating memory retrieval should be considered as coming from cortex, but that seems to be the only influence from cortex on hippocampus). It should be clarified that what cortex (the LLM) does in the model doesn't affect the evolution of what is stored/retained in hippocampus. Is this by design? Do the authors believe that this is reflective of what happens in the brain?

We believe that the model already features 'bidirectional interactions between hippocampus and cortex': one direction is the effect of hippocampal episodic memories on neocortex during consolidation, and the other is the effect of neocortical semantic memory on hippocampal episodic memory during encoding (plus the retrieval aspect you note).

The LLM (i.e. model neocortex) does affect what is encoded in the hippocampus, firstly because the gist stored is aligned with the representations of the LLM, and secondly because which details are poorly predicted depends on the current state of the LLM. To clarify how xRAG works, the projector maps from text to a single token vector which can be fed into the LLM. In other words, the projector 'translates' the input sequence into the 'language' of one particular LLM. As the LLM changes, the projector must be updated too (but we don't model this here).

Thanks for pointing out that this point was unclear in the previous version. We have tried to clarify this.

5. Besides section 2.3, consolidation is only examined through the episodic memory reconstruction tasks. Other types of tasks are needed to show that semantic knowledge is learned and that neocortex can use it to answer questions beyond simply replaying the events. Similarly, it would be interesting to test the model on more general tasks before consolidation, to study neocortex's ability to selectively extract task-relevant information to guide output generation. This could establish whether the representation serves merely as an efficient compression, or also as a flexible format that supports downstream processing in the neocortex.

Thanks for pointing out this omission. Whilst we previously considered semantic memory in the context of spatial and relational inference, it is sensible to consider semantic memory for narratives too. We have now added tests of semantic memory to the main text simulation.

We did this by asking the model neocortex factual questions about 100 stories from the dataset, e.g. 'Remember the story in which 'Jane had recently gotten a new job.' Why did Jane arrive late on her first day?'. Each prompt contains enough context to pick out a single relevant story, and the question has an answer in that story. The LLM's continuation is compared to the correct answer, and to two incorrect answers. Semantic memory is quantified as the fraction of questions for which the output is most similar to the correct answer.

The updated version of Figure 3 shows that semantic memory performance increases over the course of consolidation. Table 3 gives some example questions and answers before vs. after consolidation.

6. For the relational inference tasks in section 2.3, the model used is drastically different from the model used in previous sections. GPT-2 Medium (even though smaller than the previous model) is still quite large for simple relational inference tasks, and the idea of compression is not studied. Why? It's a bit unclear how this section fits into the larger picture of the manuscript. Was the idea simply to show that neural networks can learn structure in data ("semantic memory"), given that we're not sure how structured and generalizable the representations of the network were in the previous sections when it was processing language? How is this different from

what has previously been done in the literature, e.g., in the Tolman-Eichenbaum-Machine paper?

Yes, the purpose of these simulations was to show how these models could support the emergence of structured representations which support in-context learning. The narrative simulations use models with a lot of 'general knowledge' in their pre-trained weights, and do not fully address how this is acquired. Here we investigate how consolidation develops generalisable knowledge. These results also help to demonstrate that the same model can apply to linguistic and non-linguistic stimuli.

The reason for using a smaller model (as well as a simpler task) here is to allow more mechanistic understanding of how the model performs inference. (Note that we now also visualise the hidden representations in the family tree case.)

Whittington et al. (2020) hard-code the distinction between structure and content, and use an architecture which is specific to relational inference. Here the model receives nothing but sequences of observations, with no priors about their meaning built into the network. The model can abstract the underlying structure (a 2D gridworld or a family tree) based on only prediction error minimisation. Some improvements to the simulation make the generalisation abilities of the model more compelling than in the previous version.

In Section 2.3.1, we then develop these ideas further to explore how episodic memories contribute to novel problem solving in the model through RAG. To expand on this, working memory is limited, so the brain must selectively recall relevant experiences. In addition, problem solving can synthesise information from more than one episodic memory retrieved from hippocampus; multiple fragments of information encoded at different times can be recombined with semantic knowledge, e.g. when inferring that A is the sibling of C from the observation on one day that A is the child of B, and on another that B is the parent of C. The point of Figure 6j and k is simply to show that neither the hippocampal model nor the neocortical model in isolation can explain problem solving from recent episodic memories, and to demonstrate that the combination can combine information from *multiple* memories to draw inferences that are impossible from individual memories.

7. The authors broadly connect their work to many parts of the literature, but what is missing is a concrete comparison of their model with other published models of memory consolidation. What specifically did we learn from this model compared to what was known before, and how do its predictions differ from those of other models? Putting these results in context by relating them to previous (often simpler, and more interpretable) models would be valuable here.

We hope Table 1 addresses this.

8. In terms of writing, the language used for reporting results appears to be strongly influenced by the interpretations the authors impose on their observations. (E.g. in

section 2.2, how do we know that the information stored in the LLM corresponds to semantic memory derived from the episodic inputs?). This occasionally makes it difficult for the reader to extract objective descriptions of what was actually done and what was measured. With a more careful choice of wording, and a clearer distinction between methodology/observations and interpretation, this could be improved.

We agree that some of our attempts to explain the interpretation of the results to readers unfamiliar with LLMs etc. can make it 'difficult for the reader to extract objective descriptions'. We've tried to separate out the interpretation from the facts in several places in the paper.

Minor issues and questions:

1. What does the text embedding model and the projector in the xRAG model correspond to in the brain? Are they really part of the neocortex?

The general idea that the cortex compresses a stream of perceived stimuli to a more compact representation, from which cortex can approximately reconstruct the stimuli, has considerable support. (However, as noted above, there is some arbitrariness to the implementation of the compression aspect.)

For example, Mack et al. (2020) show that prefrontal cortex compresses away irrelevant information during learning, reducing the dimensionality of the stimuli, and Hahamy et al. (2023) show that the brain constructs compressed representations of narrative events (temporally extended segments of a story), which are reactivated at event boundaries. We've noted this in the text.

2. More explanation is needed on the generation of surprising details. Some equations could be added in the supplementary section for explanation. Are "surprising details" a set of phrases or a sentence from the story?

We have updated this in the Methods – surprising details are phrases rather than sentences.

3. For some events, the consolidated memory has a longer life span, while others have a shorter life span. Can this effect be reproduced in (and possibly predicted by) the model?

This is a very interesting suggestion - we tested this, and found the retention of memories during the 'forgetting phase' varies greatly. To recap, during the forgetting phase new episodic memories are consolidated, resulting in interference with the older episodic memories for which memory is tested.

We measured the amount of forgetting for each story as the difference between the recalled version's embedding similarity to the original at i) the beginning and ii) the end of the 'forgetting phase'.

We tested whether narrative properties of the original story predict the degree of forgetting, and found that higher concreteness, richness of detail, and specificity were associated with more forgetting. Controlling for baseline similarity, the degree of forgetting is still significantly associated with concreteness (partial $\rho=0.171$, $p=1.17e{-4}$), richness (partial $\rho=0.135$, $p=2.41e{-3}$), and specificity (partial $\rho=0.142$, $p=1.48e{-3}$). We have noted in the Discussion that how memory and forgetting differs as a function of schema congruence should be explored further.

4. Fig3(b,c): Why is the original story more similar to the reconstruction than the encoded memory at the beginning of (b) and the end of (c)?

Early in b) (before consolidation) and late in c) (after consolidation of many new memories since the consolidation of the memory in question), the encoded version is not well represented in the neocortical network. The fact that the LLM's continuation at this point resembles the original more than the encoded version may be because the process of reconstruction from gist can produce wording that is more surprising to the LLM than the original story.

5. In the priors experiment, background stories and the Bartlett story are trained together. So the background stories aren't truly previous experiences. Do the results change if the training is done sequentially?

This is a good point. Whilst jointly training on the Bartlett story and a background dataset has a similar effect to training on the background dataset *then* the Bartlett story, the latter makes more sense and avoids confusion about the meaning of 'priors'. We have changed the simulations to do this.

6. In the priors experiment (line 218), is the raw text used for fine-tuning? Or is the model fined-tuned on reconstructions of encoded stories? If it is fine-tuned on raw texts, why include the Bartlett story?

The raw text is used, for both the background texts and the Bartlett story, because otherwise it is hard to disentangle the effect of encoding and the effect of consolidation.

Sorry but we didn't quite understand the final question ('If it is fine-tuned on raw texts, why include the Bartlett story?'). In the previous version of the paper, the Bartlett story was consolidated jointly with the background dataset, and in the new version, the Bartlett story is consolidated after the background dataset is consolidated. The background dataset represents prior learning, and the Bartlett story a new experience consolidated into a network with existing schemas.

7. How would the batch size of consolidation affect forgetting? For example, for 100 memories to consolidate, does it matter if we take 2 memories per stage and 50 stages vs. 50 memories per stage and 2 stages?

This is an interesting point. We assume by batch size you mean the number of memories consolidated in one go (e.g. 50 in the forgetting simulation in Figure 3c), rather than the batch size for gradient descent in the standard machine learning literature sense (which is 1 throughout the paper).

In your example, consolidating 2 memories per stage for 50 stages (where each stage lasts for multiple epochs) would very likely result in more forgetting of the early memories than the alternative, due to classic catastrophic forgetting effects. In general, neural networks do better with interleaved training data, so a larger batch size works best as more memories are shuffled together.

We have noted in the discussion that the degree of catastrophic forgetting in this model, as in most neural networks, is unrealistic.

8. Typo: For Fig5(e) should the correct output be “ye”, rather than “o”?

Thanks, we’ve fixed this.

9. Is the model in 2.3.2 fine-tuned from the model in 2.3.1? Or is it trained from scratch? Shouldn’t the model from 2.3.1 be able to perform the task in 2.3.2 even without RAG?

The RAG simulations use the same spatial and family tree models as in the previous results. They are unable to do the task without RAG as the tasks do not contain sufficient information in the prompt to solve them with in-context learning, unlike in the previous results. (Prompts are very short, e.g. ‘xy EAST’.)

The motivation for this difference is that the previous results explore the ability to do inference based on structure learned during consolidation. Section 2.3.1 (previously, Section 2.3.2) then explores how inferences can be made across episodic memories retrieved from the hippocampus during RAG.

10. Couldn’t the distortion effect observed in Fig. 4a appear in any neural network that has learned to represent a certain input distribution and is then gradually trained on an out-of-sample input ?

Yes, other sequential generative models capable of capturing natural language would probably display the same phenomenon, but LLMs are currently the only class of model that can do so in practice. We have tried to clarify that the success of LLMs does not support the idea that the brain uses anything like ‘attention layers’.

(For example, In Spens and Burgess (2024), we show that consolidation moves a memory towards the network’s ‘priors’ with a much simpler class of model and stimuli - a variational autoencoder and a small image dataset.)

11. What are the error bars in Fig. 3? Specifically, those in panel d) and g) look tiny. What is the statistical test being performed here?

The error bars in Figures 3d and g (now e and h) give the standard error of the mean. Their small size is because both the HIPPOCORPUS dataset and the model output dataset are very large. The statistical test performed is a paired t-test (as the same story occurs in the dataset in original recalled, and retold forms). See Tables S4, SI for the stats in full.

Thanks again for all your helpful comments, which we hope have improved the paper.

Response to reviewers

Thanks very much to all the reviewers for their helpful comments, which have greatly improved the paper. We really appreciate the time you've spent on the reviews.

Reviewer #1 (Remarks to the Author):

The authors seem to have made some nice improvements in response to other reviewer comments. I continue to be supportive of this work, without any further concerns.

Thanks again for your positive feedback!

Reviewer #2 (Remarks to the Author):

The authors have significantly improved the clarity of the manuscript. From the perspective of neural activity data, I consider that the proposed model still exhibits some important discrepancies relative to neurobiological studies, e.g., memory replay. However, based on psychological data, the model proposes an interesting novel view of hippocampal-neocortical interplay during the encoding, consolidation, and retrieval of episodic memory. How these cortical regions interact at various stages of episodic memory processing has been an important topic in neuroscience, and the proposed view can enhance related future studies. This reviewer has no further comments on the manuscript.

We're very glad you found the revised paper clearer. Thanks again for your feedback!

Reviewer #3 (Remarks to the Author):

The authors have made their best attempts at addressing all the points raised by the reviewers and the clarity of the paper has much improved, but in our view its fundamental "epistemological" weaknesses remain. Except for the very positive reviewer 1, these were noted by all reviewers and expressed in complementary form. Here, for the sake of brevity, we refer to the new Table 1 and to the slightly modified Fig. 1.

Table 1 lists, in the first column, three basic questions that, we all agree, have been around for decades. In the second column it lists, as "our" hypotheses, very reasonable general ideas that we appreciate are also "theirs", but are shared with many researchers in the field. Not necessarily by all: as to the first question, for example, we subscribe to the first generic hypothesis that "SM develops through the consolidation of EM", less to the more specific one that "general knowledge can be derived by prediction error minimization on specific memories" – although the "can" would make us agree that it is indeed a possibility in some particular instances, why not? Others, who see prediction error minimization everywhere, will probably embrace the statement wholeheartedly. Other statements in the column are likewise extremely hard to disagree with, such as "consolidation mechanism produces 'approximation of approximation'" or "EM and SM combine in working memory to solve problems". The third column is not really (cognitive neuro)science but a

summary description of an LLM implementation. Fine, nobody would want to argue with it and impose on the authors a different implementation. The fourth column spells out the achievements of the implementation, which are also fine but, if we may, nothing to write home about. LLMs are capable of astonishing feats (unfortunately!), so it is hard to be impressed that they can produce “learning in non-linguistic tasks”, “a similar pattern of memory distortions to human data” or “episodic contributions to problem solving”. More telling, for us, is the fifth column, which lists the paucity of relevant/competing models. They are not so few, in fact, but perhaps the right question is: when is it that a cognitive neuroscience model becomes something more than a concise formalized summary of a set of ideas, and develops into a tool producing an original contribution? This could be in formulating predictions not derivable from the ideas alone; or in enabling simulations that allow for quantitative falsifiability not attainable with qualitative reasoning; or a mathematical formalism with eg theorems and formulas; or in other forms. Maybe developing properly formalized models in this domain has been limited by the complexity of episodic and semantic memory and their interactions, which makes any reasonable prediction verifiable in some example, real quantification difficult, and mathematical theories too complicated.

So, we are left with the cartoons of Fig.1, which are fine, but they illustrate some cognitive neuroscience inspiration for LLMs, rather than LLM contributions to understanding human memory. Curiously, the cartoon that in the original submission was coming closer to serving the latter role, ie the right half of panel d), has been expunged in the revision, as if to stay clear of any definite neuroscience statement.

Other aspects of the response to the reviews are also not convincing. For example, we asked whether the LLM used for scoring will remain accessible, and the response “we have saved the scoring data in the GitHub repo to enable the raw data to be examined if the future model changes” does not really allay our concern, of what happens once the GPT4 used for scoring is not available any more. We would not want these other aspects, however, to take precedence before the critical “epistemological” worries.

Thanks for your thoughtful comments throughout the review process.

Our aim is to take many ideas relating to multiple aspects of memory and integrate them into a single mechanistic model of the interaction of episodic, semantic and working memory for learning from, remembering and planning sequential experience. We agree that there are many epistemological difficulties involved in modelling ‘the complexity of episodic and semantic memory’. Hopefully we have acknowledged the trade-off between the complexity of behaviour one can consider and the simplicity of the model / precision of its predictions in the Discussion.

Reviewer #5 (Remarks to the Author):

We thank the authors for their detailed responses to our questions. Overall, the manuscript appears to be significantly improved. In particular, it is much clearer now what the authors wanted to achieve with this study, and we appreciate the additional results on problem solving. Motivating the paper in terms of the three hypotheses introduced at the beginning substantially improves the readability of the paper, even

though these are of course not hypotheses that are being tested here — instead, the memory consolidation properties hypothesized by the authors are very much baked into the construction of the model.

Because of this, we tend to agree with one of other reviewers who noted that much of this manuscript appears to describe an engineering effort of putting together existing model components in a way that agrees with the authors' ideas about how memory consolidation in the brain should work. That is not necessarily a bad thing in our opinion, but the question remains: What did we actually learn about the brain from this study? Or in other words, are the models presented in this manuscript more than the sum of their parts, in the sense that they teach us something unexpected?

Given the mix-and-match, black-box modeling approach of this manuscript, it's unclear whether we have any right to expect concrete answers to these questions, but we believe that the construction and characterization of these models nevertheless constitute an achievement that many readers will be interested in.

Thanks for your feedback!

This review was conducted jointly with a junior researcher in our group. Below are some additional comments/questions from them:

1. In the rebuttal letter, the authors mention that surprising details they use are phrases but not sentences. However, in Table S1, the surprising details are shown as a sentence in each example. Also, Fig 3a shows that one detail is around 10 tokens, so the detail is most likely a sentence rather than one phrase.

The 'surprising details' extractor splits stories into clauses / phrases, but it can leave a whole sentence intact when there is no comma or coordinating conjunction, as is often the case in the simple English of the ROC Stories dataset (e.g. in 'The dentist tells her she has 6 cavities' in Table S1). The splitting into clauses / phrases uses a standard method from the spaCy Python library for natural language processing. We've clarified this in the Methods, as we agree our current description is ambiguous - thanks for spotting this.

2. In section 2.1.1 and section 4.2.1, the authors explain that they select phrases as surprising details by comparing perplexities on inputs of format: [gist token] + [tokens for phrase p]. If we understand it correctly, the perplexity measures the chance that the LLM generates phrase p right after the gist token. But a high perplexity doesn't necessarily mean that information about p is missing from the gist token -- perhaps p is just supposed to be generated some tokens away from the gist token. The detail in example 1 of Table S1 still has some overlap with the content stored in the gist vector, so perhaps it would be better to rephrase surprising details as supporting details.

This is an interesting point – you're right that the perplexity may be greater for phrases that occur later in the story, i.e. our 'surprise' metric may really reflect a combination of surprise and position in the story. We also agree that, since the perplexity of each phrase is evaluated as a whole, phrases may be marked as 'surprising' because some subset of tokens within them is surprising to the model.

We’ve noted this in the Methods.

3. How accuracy is calculated in Fig 3d is a bit strange. Since the correct answer is known, why not directly use it to check the model's answer (perhaps use gpt-4o for automation)? Why additionally introduce two incorrect answers and then compare the distances in the embedding space? The accuracy by the authors' method could be heavily influenced by how incorrect answers are sampled.

Embedding similarities are standard for evaluating the correctness of question answering as this is simpler and more convenient to reproduce than using, say, the OpenAI API. (The model’s answers are free-form and may be correct despite not matching the exact answer string. A complex prompt would need to be provided to an LLM to clarify the criteria for a correct answer, so we thought it was easier to avoid.)

The ‘multiple choice’ metric was used because the cosine similarity between the model’s answer and the true answer is hard to interpret (a gradual increase in cosine similarity with consolidation could be consistent with the model never answering a question correctly), whereas we thought the ‘multiple choice’ fraction correct was more intuitive. The much lower baseline performance at the start of consolidation indicates that the distractor answers are reasonable. We’ve clarified this rationale in the Methods.

4. Figure 5e in line 269 should be Figure 5f.

Thanks, we’ve corrected this.

5. The experiments on content distortions (section 2.2.1) exclude the compressive retrieval process (use the original Bartlett story for consolidation), but the experiment on structure distortions (section 2.2.2) include it. Why such a difference? In section 2.2.1 the authors mention that they want to isolate the effect of consolidation from encoding, but the same could happen for experiments in section 2.2.2. Since it seems that a major point of this paper is compressive retrieval-augmented generation, I believe it would be better to use the full framework throughout the paper.

We agree that the cleanest version of the approach would use the same compression method throughout. However, the xRAG weights we use are pre-trained specifically to map text embeddings into the token embedding space of Mistral-7B; we would need to train a new compressor for fine-tuned models. For this reason, we use the available xRAG model where it is applicable, and simplify the pipeline in other experiments. In Section 2.2.2, the existing text xRAG pipeline can be applied directly to the story stimuli, so we include the full compression pipeline there. In Section 2.2.1, different LLMs are trained with different topics as a ‘prior’, and would thus require custom compressors.

6. Similar to the previous point, experiments in section 2.3 don't involve compression of input sequences. To make the experiments more consistent throughout the paper, a potential improvement would be developing a custom xRAG model that

compresses a sequence sampled from a spatial graph or family tree to a single token.

We agree that the Section 2.3 spatial / family tree graph experiments are simplified in the same way as the Section 2.1 results. These simulations were intended to focus on whether neocortical consolidation can extract reusable sequential structure from replayed experience, using smaller GPT-2 models rather than Mistral. We agree that a natural extension would be to train a custom xRAG-style model to compress spatial / family tree sequences into the token space of our GPT-2 model, so that random walks can be compressed into a single learned token plus residual details. That would make the framework more consistent across narrative and structural inference experiments, and we have noted this as a possible future direction.
